# Prehistoric pathways to Anthropocene adaptation: Evidence from the Red River Delta, Vietnam

**DOI:** 10.1371/journal.pone.0280126

**Published:** 2023-02-08

**Authors:** Ryan J. Rabett, Risa Morimoto, Thorsten Kahlert, Christopher M. Stimpson, Shawn O’Donnell, Nguyen Thi Mai Huong, Bui Van Manh, Rachael Holmes, Phạm Sinh Khánh, Tran Tan Van, Fiona Coward

**Affiliations:** 1 Archaeology & Palaeoecology, School of Natural & Built Environment, Queen’s University Belfast, Belfast, United Kingdom; 2 Institute for Hellenic Culture & the Liberal Arts, The American College of Greece, Athens, Greece; 3 Department of Economics, School of Oriental and African Studies (SOAS), University of London, London, United Kingdom; 4 Centre for Geographic Information Science and Geomatics, School of Natural & Built Environment, Queen’s University Belfast, Belfast, United Kingdom; 5 Oxford University Museum of Natural History, Oxford, United Kingdom; 6 Department of Geography & Environmental Sciences, Northumbria University, Newcastle Upon Tyne, United Kingdom; 7 Vietnam Academy of Social Sciences, Institute of Archaeology, Hanoi, Vietnam; 8 Department of Tourism, Ninh Bình City, Ninh Bình Province, Vietnam; 9 School of Geography, Geology & the Environment, University of Leicester, Leicester, United Kingdom; 10 Tràng An Landscape Complex Management Board, Ninh Bình City, Ninh Bình Province, Vietnam; 11 Vietnam Institute of Geosciences & Mineral Resources, Ministry of Natural Resources & Environment, Hanoi, Vietnam; 12 Department of Archaeology, Anthropology & Forensic Science, Faculty of Science & Technology Bournemouth University, Poole, Dorset, United Kingdom; Zoological Survey of India, INDIA

## Abstract

Over the past twenty years, government advisory bodies have placed increasing emphasis on the need for adaptive measures in response to the effects of human-induced climate change. Integrated Assessment Models (IAMs), which incorporate macroeconomic and climate variables, feature prominently in advisory content, though they rarely draw on data from outside strictly constrained hypothetical systems. This has led to assertions that they are not well-suited to approximate complex systemic human-environment processes. Modular, interdisciplinary approaches have offered a way to address this shortcoming; however, beyond climate records, prehistoric data continue to be under-utilised in developing such models. In this paper we highlight the contribution that archaeology and palaeoecology can make to the development of the next generation IAMs that are expected to enhance provision for more local and pro-active adaptations to future climate change. We present data from one of Southeast Asia’s most heavily developed river deltas: the Red River (Song Hong) Delta, in Vietnam and localised analysis from the Tràng An Landscape Complex World Heritage Site, on the delta’s southern margin. Comparison is made between Shared Socio-economic Pathways (SSP) 5–8.5 and SSP2–4.5 emission projection models and the Mid-Holocene inundation of the Red River Basin. We highlight the value to taking a scientific long view of coastal evolution through an illustrative set of eight research foci where palaeo-data can bring new and localised empirical data to bear on future risk management planning. We proceed to demonstrate the applicability of palaeoenvironmental, zooarchaeological and historical evidence to management and the development of sustainable conservation strategies using Tràng An as a case study. In so doing, we further highlight the importance of knowledge exchange between scientific, corporate, non-governmental, local, and state stakeholders to achieve tangible results on the ground.

## Introduction

Adaptive measures form an increasingly critical part of global responses to human-induced climate change [1–7]. The development of such solutions is enhanced significantly by considering contemporary evidence within a broader temporal context, especially one that encompasses more than the last couple of centuries [8–10]. Palaeo-climate records obtained from ice and sedimentary cores already contribute important data to global change science [4, 11–13]. However, while the potential for wider use of deep-time records of environmental change and human activity has been highlighted repeatedly [14–27], their relevance to mainstream climate and ecological advisory and policy literature is still not widely recognised. Here we show where the incorporation of prehistoric evidence can help inform the next generation of Integrated Assessment Models (IAMs) that seek to establish local pathways to sustainable infrastructure management, particularly in the context of vulnerability to coastal inundation. Demonstrating the value to be found in palaeo-data requires more than simply presenting the science, however. Archaeologists and palaeoecologists must identify and develop ways through which their work can inform practice [14, 28–31]. At the same time, there must be multi-stakeholder buy-in from outside of these disciplines. In this paper we illustrate how both these objectives can be achieved.

### Adaptation and Integrated Assessment Models (IAMs)

IAMs incorporate macroeconomic and climate models in order to simulate alternative future climate scenarios resulting from different policy actions [32, 33]. Since the 1990s they have come to dominate simulations of the impact of climate change, though traditionally, the focus has been at the level of individual economic sectors (e.g., agriculture or forestry). The majority of original IAMs also rarely incorporated adaptation as a variable [34]. While the Policy Analysis of Greenhouse Effect (PAGE) model was one exception, even this treated adaptation as a variable set by the modeller [32, 35, 36]; indeed, adaptation as part of systemic response to climate change was not formally defined in respect to such models until 2001 [2]. These factors inevitably reduced the effectiveness of decisions about climate adaptation [37], and it is now widely accepted that greater consideration of temporal and spatial scales and interdependencies within human and environmental adaptive systems is required [37–39]. Even now though the scale of uncertainties and constraints involved presents difficulties to estimating the impact of adaptation variables on modelled outcomes [37]. This situation is exacerbated by the fact that state-of-the-art climate models may be underestimating the rate and extent of change by weighting calibration towards processes that are discernible through observational records at the potential expense of impacts, including transformational ones, from feedback mechanisms that operate over longer time spans [40, 41]. Greater interrogation and incorporation of palaeo-data is required to rectify this.

More recent IAMs have proven capable of simulating passive adaptation in the form of endogenous market responses to climate-induced changes (e.g., reductions in rain-fed crop production induced by decreased productivity of land due to lower precipitation). However, simulating the outcomes of pro-active (i.e., planned, or anticipatory) measures also remains a challenge. IAMs can capture the trade-off between future damages and the mitigating effect of current defensive adaptation expenditures via a ‘damage function’ (i.e., the penalty for environmental degradation on production), but do so only imprecisely. For these reasons, simulations continue to be employed within quite strictly controlled sets of conditions or have assumed that the residual damages from climate change are minimised [42–44]. Thus, as the timing and magnitude of the impacts of climate changes become increasingly difficult to predict the further forward in times one goes, so the returns on investments in adaptative measures intended to protect against future impacts also become increasingly difficult to predict. Despite being a mainstay of widely circulated and influential outputs from government advisory bodies, the capacity of IAMs to simulate the complexities of real-world conditions is now under considerable scrutiny [25, 37, 45–48].

Recognition of these weaknesses has driven efforts to combine data at finer scales or from outside tightly constrained economic parameters [49–51]. For example, the Dynamic and Interactive Vulnerability Assessment (DIVA) tool has incorporated natural- and social science-derived data [52]. Increasingly sophisticated iterations of DIVA’s modular approach have been developed across a range of frameworks at different scales to simulate future risk to global and regional coastlines [53–60]. However, the incorporation of adaptive measures into these goal-oriented models, even of a more spatially resolved type, continues to focus on hypothetical least-cost approaches towards achieving optimal management at broad scales that, arguably, do not sufficiently consider local conditions or the potential impact of non-linear dynamics, both of which are now seen as key to future conditions [61–64]. To improve the reliability of forecasts and specific local outcomes of sea level rise, it is now recognised that the next generation of Climate Change Impact, Adaptation & Vulnerability (CCIAV) models will need a less abstract and more bottom-up approach to adaptation [31, 37]; one that balances aggregated global scale IAMs with specific disaggregated local landscape scale circumstances to deliver effective strategies [65–68]. Part of that revision includes a growing consensus that projected outcomes of sea level change must take greater account of the effects of long-term coastal processes and evolution [68–76]. In the following sections we illustrate the value of such a perspective, drawing particularly on the results of fieldwork undertaken as part of the SUNDASIA Project (2016–19) in the Tràng An Landscape Complex World Heritage Site, Ninh Binh Province, Vietnam.

### Future and past inundation of the Red River Delta

By 2100, almost a third of coastal lowlands at risk from a predicted *c*. 1 m rise in sea level will be in tropical Asia [77], with Vietnam ranking as one of the most vulnerable nations [78–80]. Currently, *c*. 70 percent of the country’s 93 million people live along its 3200 km coastline and ‘mega-deltas’ [81], exposing significant sections of the country’s economic activity to sea level rise impacts [82–84]. The significant variability in regional and local effects from global mean sea level change requires spatial and temporal refinement when assessing future coastal conditions [69, 85–88]. Localised fluctuations in prehistoric sea level are still not fully understood in Southeast Asia [89–91]; however, multiple high-quality datasets are now available [92–100].

For this paper, we created future coastline models calculated from Climate Central’s re-worked Shuttle Radar Topography Mapping Digital Surface Model (SRTM DSM) [101, 102] and sea level projections available via the NASA IPCC AR6 Sea Level Projection Tool (SLPT) (*see* Methods 1) [103–105], localised to Vietnam’s Hon Dau National Sea Level Datum. From these data we created a low resolution SRTM-derived DSM that simulated two Shared Socio-economic Pathways (SSP) at radiative forcing levels 5–8.5 –specifically, at medium and low confidence levels–and the ‘most likely’ (SSP2–4.5 medium) emissions scenario to model how rising seas may affect the Red River Delta (RRD). The chosen predictive scenarios use values from the 50^th^ quantile for the years 2050, 2100 and 2150. The DSM is adjusted for skewed elevation due to the presence of vegetation and built features [101, 102].

**Fig 1:**
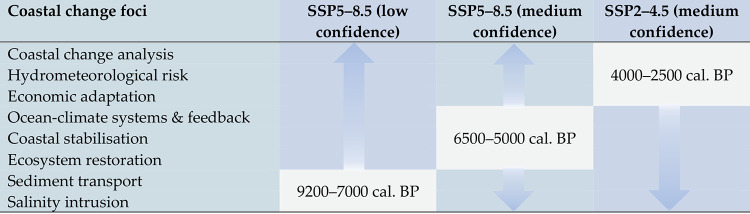
Examples of coastal change foci of attention to which palaeo-data can make significant contributions in the context of modelling human and landscape responses to future sea level rise projections under emissions scenarios SSP5–8.5 and SSP2–4.5. Highlighted time windows represent periods of focus herein; arrows indicate wider applicability of datasets.

**Fig 2:**
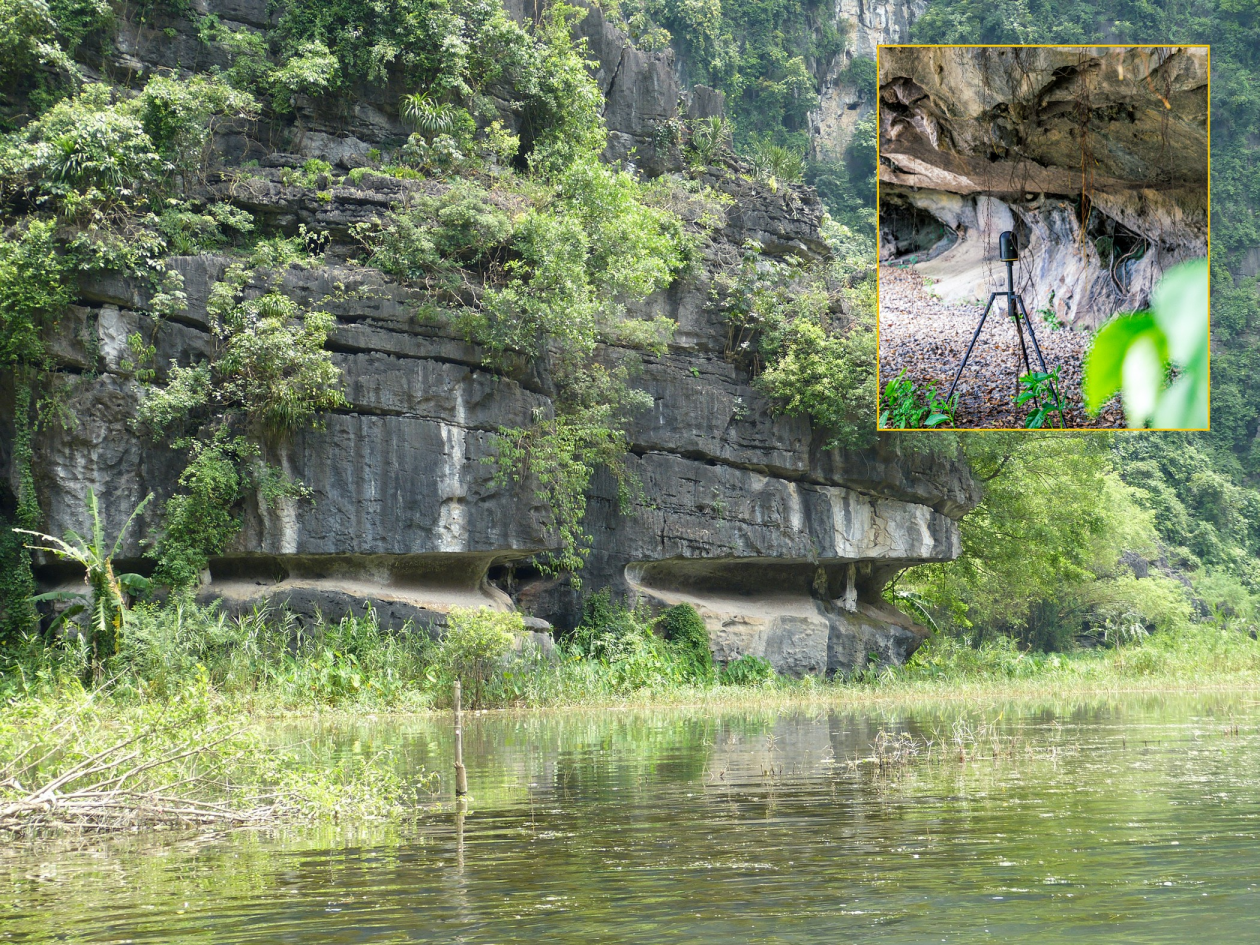
Well-preserved corrosion notches in Tràng An, such as these in the Tam Coc-Dich Dong part of the property, reveal separate phases of sea level stability during the Mid-Holocene marine transgression (8000–4000 cal. BP). Notch locations and elevation data were recorded using either a Leica GS15 nRTK (network Real Time Kinematic) GNSS (Global Navigation Satellite System) receiver, and Leica TS06 total station, and later (in 2022) also a Leica BLK360 imaging laser (see inset). (Main photo: Ryan Rabett, inset photo: Thorsten Kahlert).

As a first step towards integrating greater spatial-temporal resolution into models of coastal change, we reference three time-intervals (9200–7000 cal. BP, 6500–5000 cal. BP & 4000–2500 cal. BP) during which palaeo-coastline configuration in the RRD was broadly compatible with the SSP5–8.5 and SSP2–4.5 sea level scenarios. These are not intended to represent a direct analogue to modern or projected conditions. Each time-interval is intended to provide a spatially controlled starting point from which field-based prehistoric evidence can aid foci affecting coastal change and, by extension, the development of responsive resource management, and flood mitigation strategies. Eight such foci are discussed (Fig 1), though this is not intended to be an exhaustive list. For the purposes of this paper, where our goal is to highlight the potential utility of palaeo-analysis at a sub-regional and local scale, we have not attempted a systematic segment-by-segment assessment of the deltaic coastline (an approach taken, for example, by Fan et al. [106] and Ve et al. [107] in their historical time-series analysis of the RRD). However, we envisage that this could mark a logical next stage. Palaeo-coastline models are drawn from the literature and are based primarily on sediment core lithology, composition, and biological proxies as relative sea level indicators. In addition to these data, one of the sources, Hoang et al. [108], incorporated geomorphological proxies and shallow seismic sections into their model. Our own data from Tràng An [100] (*see* Methods: 1) similarly, relies on geomorphological proxies, particularly corrosion notches (Fig 2), supplemented by archaeological and coring data [109].

**Fig 3:**
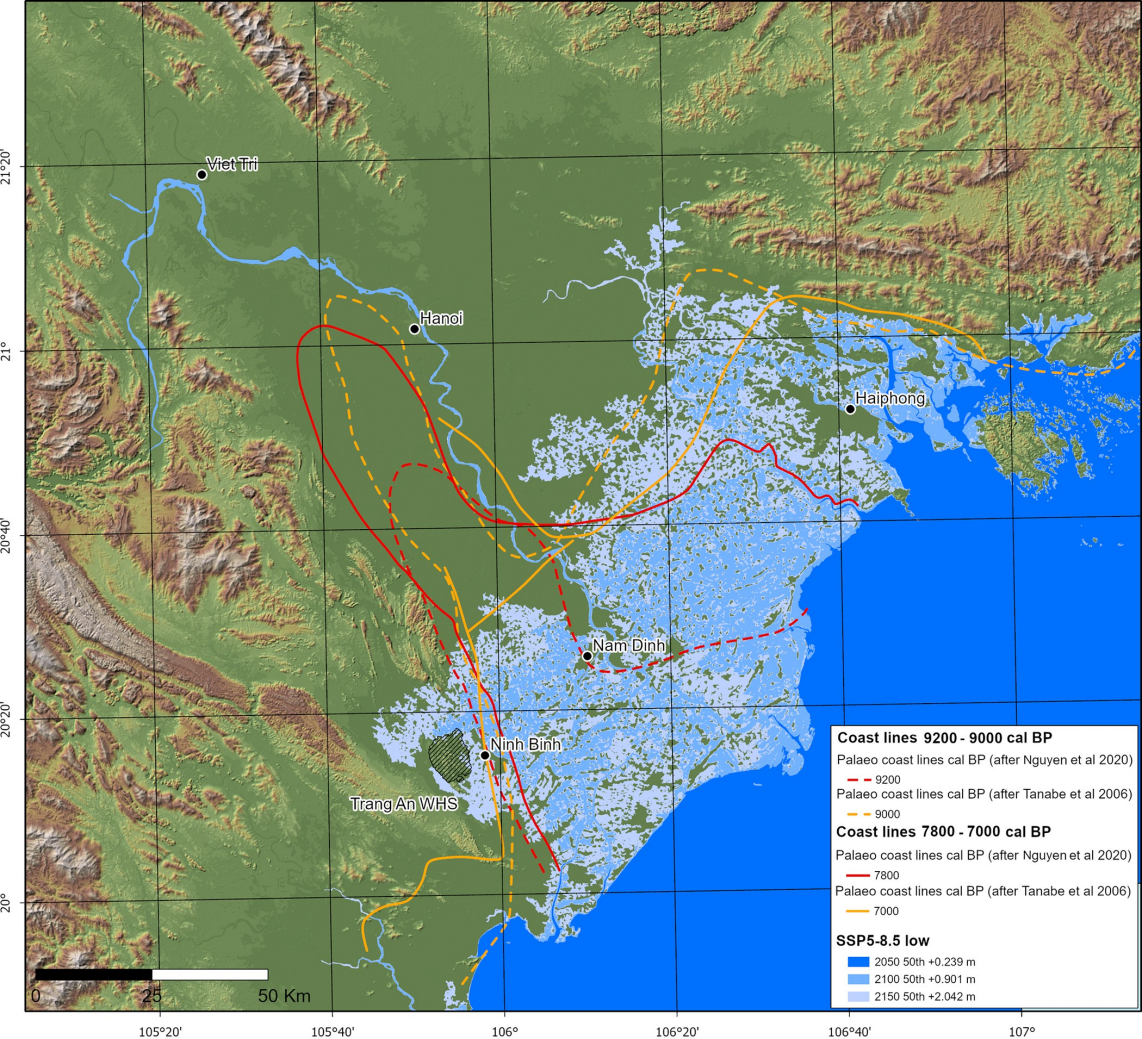
SSP5–8.5 (low confidence) sea level rise models for the years 2050, 2100 and 2150, contrasted with published early to Middle Holocene coastline models. The Tràng An Landscape Complex World Heritage Site is highlighted. All SSP models were generated by Thorsten Kahlert exclusively for this paper using Climate Central’s re-worked Shuttle Radar Topography Mapping Digital Surface Model (CoastalDEM® courtesy of Climate Central https://go.climatecentral.org/coastaldem/). Projected sea levels were obtained via the NASA IPCC AR6 Sea Level Projection Tool (https://sealevel.nasa.gov/data_tools/17) (Image: Thorsten Kahlert).

Fig 3 presents the SSP5–8.5 low confidence and current worst-case set of scenarios, for predicted sea level rise, including a mean sea level of +0.90 m by 2100. For this scenario, we found that the early transgressive phase leading up to the Mid-Holocene (approximately, 9200–7000 cal. BP) provides a salient point of comparison in terms of coastal configuration. Assuming that the RRD is not subject to compensating subsidence (i.e., where subsidence is balanced by sediment supply), this was a period when the lower delta, particularly in the vicinity of the Red River Deep-Seated Fault, likely lay 20–40 m below modern ground level. This created initial conditions for extensive inundation despite sea levels still being 10–30 m below those of today. Reference to this interval can assist modelling for worse-case scenarios that local policymakers are now considering [110]. Palaeo-data from 9200–7000 cal. BP with respect to sediment transport and salinity intrusion are instructive.

#### Sediment transport

Extending the reference timeframe for precipitation into the more remote past is now recognised as essential to developing robust hydraulic models for flood defence, flood mortality prevention, and water-dependent infrastructure [73, 111]. Although subject to long debate about its inter-regional asynchronous nature [112], the Holocene Climatic Optimum (HCO) of the East Asian Summer Monsoon (EASM) represents a strong, sustained, and possibly unstable interval [113] that can provide a helpful calibration for simulations of future precipitation change [114].

Palynological evidence in the vicinity of the northern South China Sea place the HCO 9500–8000 cal. BP (Huguangyan Maar Lake) [115], 9000–6000 cal. BP (GLW31D core, northern continental shelf) [116], 9000–7000 BP (U-Th) from the Dongge Cave speleothem record [117], and 8000–7000 BP (Chongqing, southwest China, and synthesised record) [113]. Coral records from Sanya, Hainan Island [118] also suggest summer sea surface temperatures (SSTs) may have peaked at up to 2°C higher between 6496 and 6460 BP (U-Th) than those reported for the second half of the 20^th^ Century. With appreciably heightened levels of precipitation accompanying the optimal EASM (20–30% above present values, based on evidence from further east–Xinjie, in the lower Yangtze Valley, [114]) this likely resulted in increased surface run-off and sediment transport into regional river systems [119]. Such conditions are reflected in a 9.11 m core extracted from the northeast margin of the Tràng An massif (20° 17’7.01” N, 105° 54’ 21.59” E) from which almost 7.5 m of deposition accumulated over a period of *c*. 500 years, from 7948–7720cal. BP (UBA-25530) to 7576–7458 cal. BP (UBA-25527) [120]. Allowing for differences in evolutionary history between deltaic systems, the average sedimentation rate here of 15.16 mm/yr is comparable to the high rate recorded in the Pearl River deltaic basin (11.8–15 mm/yr), and in other Asian deltas for this period [121, 122]. These data therefore present reference potential for models exploring the impact of the anticipated increase in heavy precipitation during this century [5].

#### Salinity intrusion

The relationship between climate change variables and groundwater is still poorly understood [123, 124]. Under current conditions, salinity intrusion is greatest during the dry winter monsoon (December–April), when low river discharge means that tidal action from the South China Sea can raise groundwater salinity for tens of kilometres inland [124–126]. If exacerbated by drought conditions from strong El Niño Southern Oscillation (ENSO) events, this intrusion can reach much farther. For example, the 2015/16 ENSO coincided with intrusion up to 90 km inland in the Mekong delta [127]. Studies examining the impact of drought in the RRD are scarce, though the effects to salinity intrusion are expected to be similar [128], and with ENSO intensity predicted to increase this century [129], palaeo-records stand to make a valuable contribution to our understanding of its Holocene evolution and impacts [130].

Currently, sea level rise is expected to accelerate saltwater intrusion into the RRD’s already heterogenous aquifer system (comprising zones of fresh and salt water), causing significant damage to the delta’s crucial agricultural sector [123, 124, 131]. As Larsen et al. [74] explain, in the shallow Holocene (unconfined) aquifer, palaeo-saltwater extends up to 25 km inland (at maximum chloride levels of 19.6 g/l), decreasing to *c*. 10 g/l (brackish) at 50–60 km from the coast. As 4 g/l is the maximum tolerance for wet rice cultivation [132], deviation above this value will likely decrease yields and increase the need for water management [126]. Salt intrusion gates on the Red River, Tra Ly River and Hoa River [133] already offer hard infrastructure solutions but pose issues for ecological services [125]. If groundwater chloride levels in coastal aquifers take up to 40–50,000 years to adjust to rapid sea level change, as Larsen et al. indicate [74], palaeo-data will be directly relevant to refining salinity intrusion data.

**Fig 4:**
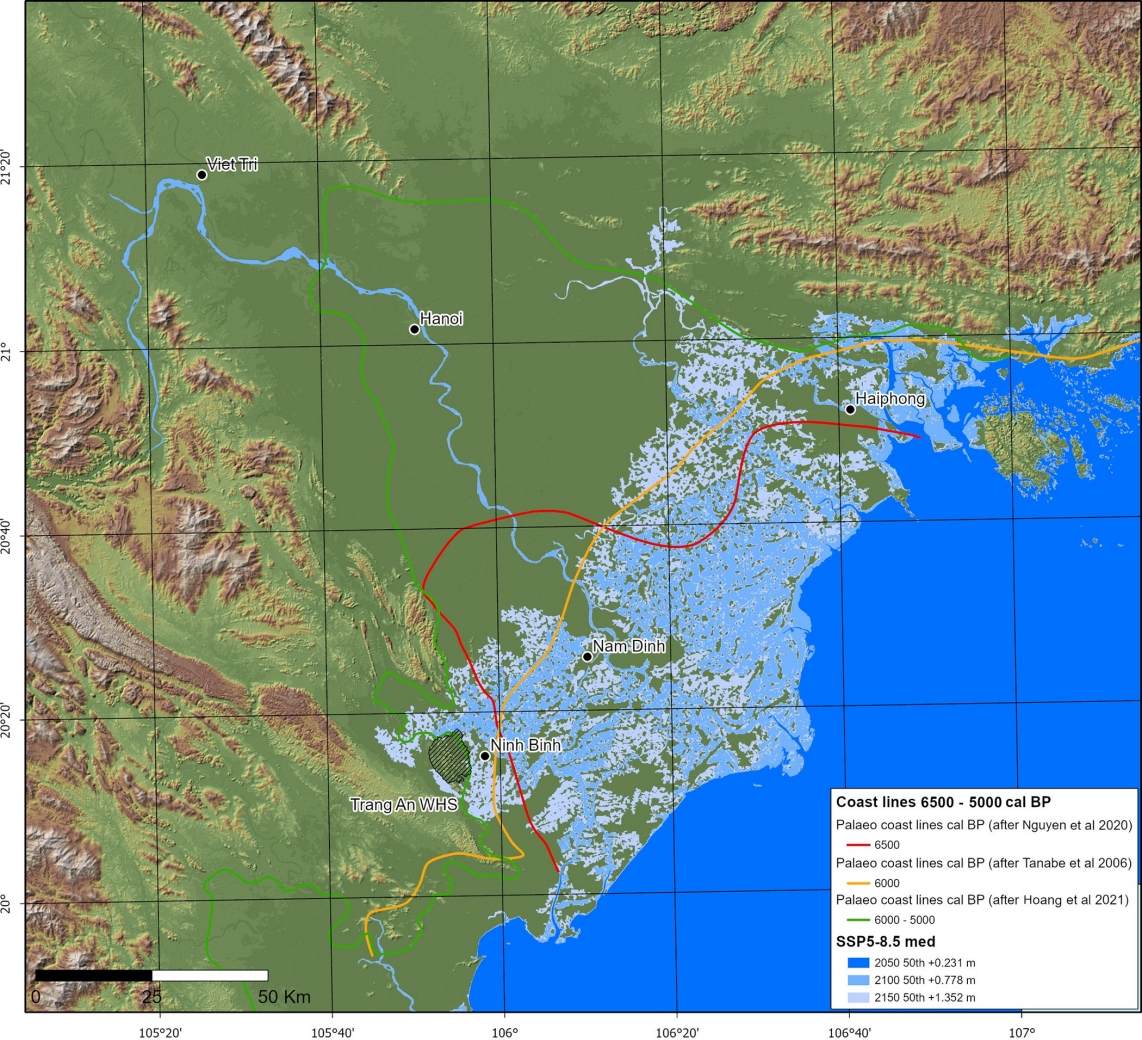
SSP5–8.5 (med. confidence) sea level rise models contrasted with Mid-Holocene coastlines. CoastalDEM® courtesy of Climate Central (https://go.climatecentral.org/coastaldem/). Projected sea levels were obtained via the NASA IPCC AR6 Sea Level Projection Tool (https://sealevel.nasa.gov/data_tools/17) (Image: Thorsten Kahlert).

Fig 4 illustrates the SSP5–8.5 medium confidence forecasts that still highlight the scale of inundation risk to the lower reaches of the RRD, equivalent to +0.77 m by 2100. Against this medium likelihood scenario, we found correspondence in coastal configurations dating to the time during and immediately after the Mid-Holocene high stand of 6800–6000 cal. BP [116], with the exception of retrodictive modelling by Hoang et al [108]. Palaeoenvironmental data from this period can help guide the modelling of ocean-climate systems and feedback loops, coastal stabilisation, and ecosystem rehabilitation.

#### Ocean-climate systems and feedback

The Mid-Holocene interval offers detailed insights into the relationship between marine inundation and climate. For example, speleothem evidence from Wuya Cave on the southwest margin of the Chinese Loess Plateau [134], supported by more proximate regional data (such as coring from the Huguangyan Maar Lake on the Leizhou Peninsula, China [135]), point to a marked transition in climate regime from more stable to more chaotic fluctuations (at millennial, centennial, and decadal scales) superimposed on a weakening (orbital scale) EASM. This shift has been linked to strengthened and increasingly variable ENSO activity from *c*. 6600 BP (U-Th) [136], around the time of the high stand. With continued uncertainty about projecting ENSO activity and impacts into the 21^st^ Century [137, 138], exploration of such links through palaeo-data provides a valuable route to more robust predictive models.

#### Coastal stabilisation

Hard infrastructure solutions, such as river dike systems, reservoirs, and flood diversion structures, feature prominently in efforts to assess and mitigate flood hazards and other projected erosional impacts from sea level rise [80]. There is also a long tradition of mangrove rehabilitation programmes in Vietnam. However, in the current drive to employ natural solutions to parallel, if not replace, the use and maintenance of hard infrastructure, the response of mangroves to sea level rise is still a matter under investigation. Based on current modelled expectations, a rate of sea level rise over 6.1 mm/year (i.e., that expected under the Representative Concentration Pathway (RCP)8.5 scenario for 2050) may exceed the tipping point at which mangroves are able to build vertically through sediment accretion [139, 140]. Hydrological and microclimate conditions affecting the prehistoric establishment and long-term persistence of back-mangrove forest have been reported for the Tràng An massif [100, 109]. This record runs contra to the wider deltaic observed trend for mangrove decline during the 6500–5000 cal. BP interval [141–145] and is explored in a later section of this paper.

#### Ecosystem restoration

This can be considered in relation to terrestrial habitats, particularly where these exhibit potential for long-term stability (see herein) but is equally applicable in relation to marine settings. For example, the dating of reef growth and die-back off the southern coast of Hainan Island (Luhuitou reef, Sanya) includes marked growth *c*. 6700–4000 BP (U-Th), linked to the Mid-Holocene high stand under conditions broadly similar to those expected by 2050, and demonstrating future coral refugium potential in the northern South China Sea [146].

**Fig 5:**
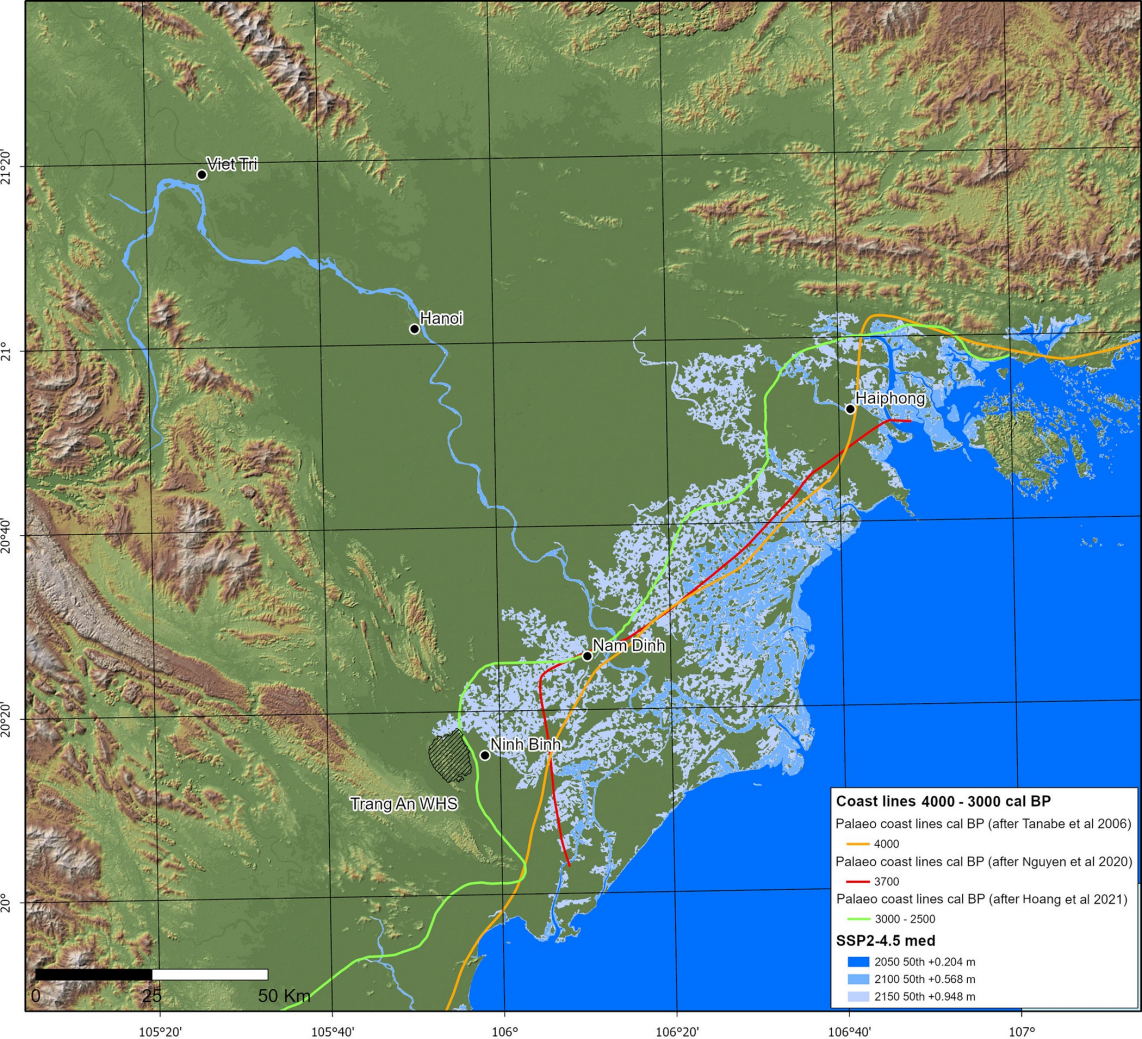
SSP2–4.5 (med. confidence) sea level rise models contrasted with Mid- to Late Holocene coastlines. CoastalDEM® courtesy of Climate Central (https://go.climatecentral.org/coastaldem/). Projected sea levels were obtained via the NASA IPCC AR6 Sea Level Projection Tool (https://sealevel.nasa.gov/data_tools/17) (Image: Thorsten Kahlert).

The most likely of the future emissions scenarios (SSP2–4.5) and the inundation risk this poses for the RRD are presented in Fig 5 for the years 2050 (+0.20 m), 2100 (+0.57 m) and 2150 (+0.95 m). Note, these values do not account for uplift/subsidence due to tectonic or human-induced factors (e.g., groundwater extraction). We found that SSP2–4.5 predictions exhibited correspondence to hindcast models of coastal conditions 4000–2500 cal. BP and, in this case, particularly that of Hoang et al. [108]. These models and associated data are well-positioned chronologically, and in terms of the substantial volume of evidence available, to complement existing analysis of coastline change, hydrometeorological hazard prevention, and in the development of economically adaptive strategies.

#### Coastline change analysis

The assessment of coastal morphological change over intermediate timescales (months to decades) is vital to effective coastal management. This is, for example, recognised in the time lag and the assignment of cause to changes in sediment discharge in the Red River following construction of the Hoa Binh Dam [125, 147, 148]. Meanwhile, at a centennial-millennial scale, the relationship between sea level change and the balance of sedimentation is there to be explored [149]. Palaeoenvironmental and archaeological datasets from the last 4000 years extend the reach of growing interest in long-term delta progradation via palaeo-geographic, historical cartographic, remote sensing, and geological studies [75, 106, 150, 151], and the extent to which factors such as, climate and heightened storm-surge frequency, human impact, and mangrove distribution influence the sedimentation balance.

#### Hydrometeorological hazards

Karst systems are particularly susceptible to hydrological disturbance and palaeoenvironmental change [152]. Such susceptibility requires close monitoring to predict and alleviate impacts from future extreme weather events, flooding, and landscape erosion. Data describing how hydrological, sedimentary, geomorphological, and subsurface processes controlled the movement of water during past periods of extreme climate can support these efforts. For example, sedimentary records from the north South China Sea, such as the Pearl River estuary and coastal dune deposits on Hainan Island [153, 154], document heightened typhoon-like activity 3000–2700 cal. BP, coinciding with increased sea surface temperature, ENSO, and storm-surge events. Sedimentological archives can help explain the complex evolution of Holocene typhoons [154, 155], the impact of tsunami [156], and how the processes controlling water movement interact across these events. Given the perceived future risk from hydrometeorological hazards to agriculture and infrastructure [157], such data stands to reduce the level of predictive uncertainty [136].

#### Economic adaptation

With localisation seen as a necessary balance to adequately model variability in response to sea level change [68, 69], efforts are under way to downscale DIVA models to include higher-resolution segmentation units of coastal areas and multiple dimensions of spatial analysis (including extending those units inland) in order to achieve more locally explicit, realistic, and relevant measures [59, 60]. Taking the area around Ninh Binh and Nam Dinh in the southern RRD, as illustrative, lithological evidence shows rapid sedimentation 4000–2500 cal. BP, in environments that correspond to a delta front platform [96, 108, 141, 145]. Macro-botanic and pollen records indicate the presence of true mangrove taxa early in the local depositional sequence, later replaced by an increasing abundance of back mangrove elements as emerging intertidal flats were colonised [141, 145]. Palynological evidence from after 3340 cal. BP shows a sharp increase in non-arboreal pollen, dominated by Gramineae (potentially including the main wet rice species, *Oryza sativa*) Araceae and Gesneriaceae, but also secondary forest, and upland cultivated taxa linked to increased human activity [143]. Such habitat change, in combination with evidence of settlement–in this case focused on substantial levees (3–8 km wide and 2–5 m above the surrounding landscape) along the Day River [142]–provide baseline information on human adaptive response to newly reconfigured and inundated landscapes.

**Fig 6:**
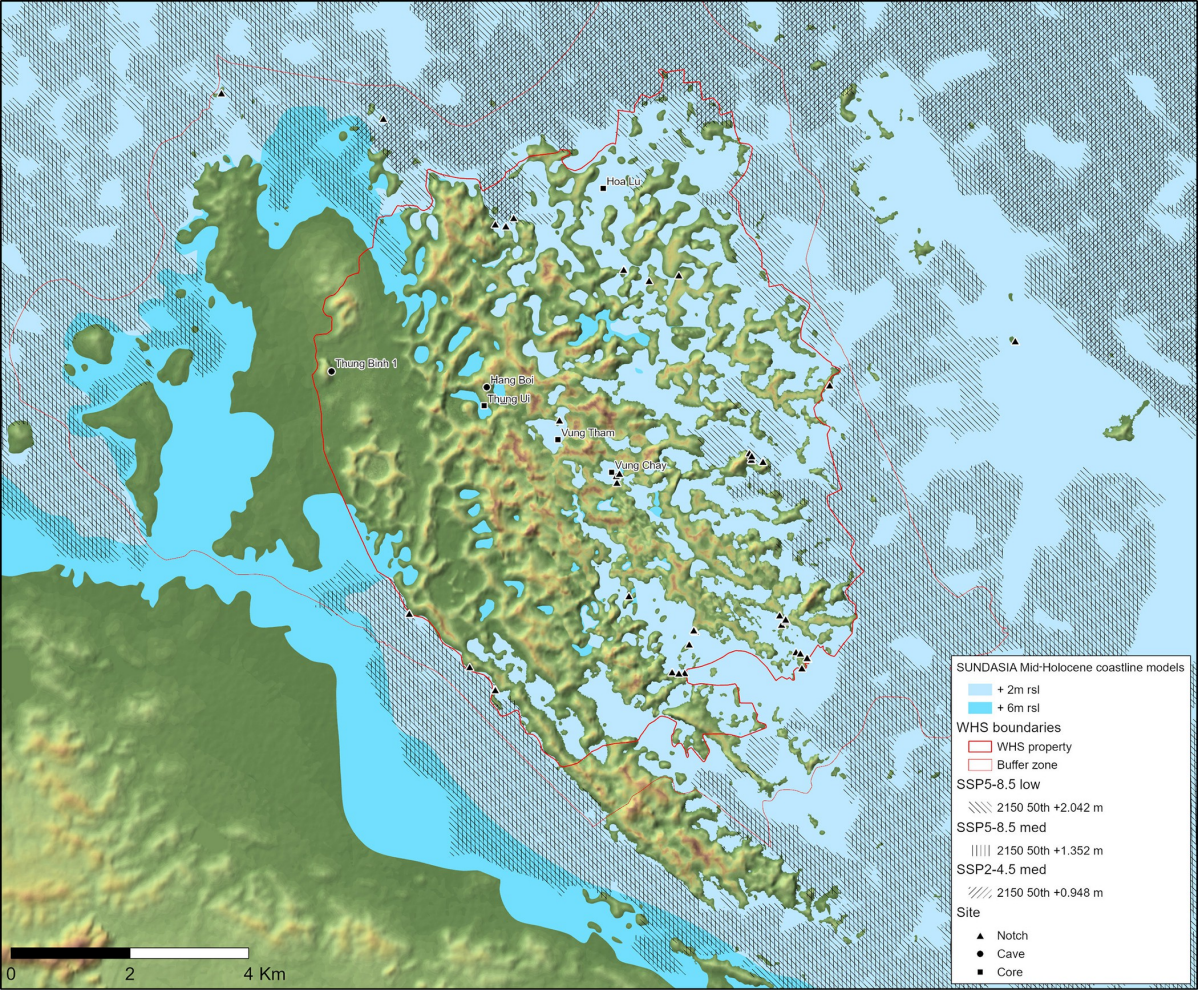
The Tràng An massif showing reconstructed min./max. elevation estimates for the local Mid-Holocene coastline [100] and projected SSP5–8.5 (low) SSP5–8.5 (med.) and SSP2–4.5 (med.) scenarios for the year 2150 (topographical base map derived from SRTM 1 Arc Sec DEM, courtesy of USGS / NASA: https://doi.org/10.5066/F71835S6). (Image: Thorsten Kahlert).

Under all of the projected emissions scenarios in this section, the Tràng An massif will become coastal by 2150 (Fig 6). This makes it an excellent ‘anchor-point’ [100] location to examine how palaeo-data can assist in creating locally attuned flood risk mitigation and conservation strategies. In the following sections we use palaeoenvironmental (mangrove), vertebrate zooarchaeological and zoological evidence from Tràng An, and highlight the critical importance of on-the-ground multi-stakeholder involvement, to spotlight how this can be achieved.

### Establishing resilient centres of mangrove forest

Southeast Asia’s mangroves are considered globally to be the most species-rich [158]. Their sustainable management, particularly in relation to impacts from sea level rise, stands to benefit significantly from a deep-time perspective. Highly specialized forests occupying the inter-tidal zone [159], mangroves provide numerous ecosystem services critical to human adaptation to climate change. The importance of ecosystem services they provide is recognised by policy mechanisms such as Payment for Ecosystem Services (PES) schemes [160] designed to ensure returns on investment into such solutions (e.g., UN-REDD+ [161] and Vietnam’s national Payments for Forest Environmental Services [162]). Mangroves are key storers of ‘blue carbon’ [163] and represent one of the planet’s remaining ‘irrecoverable carbon’ reserves [164]. They also provide nurseries for species that underpin socio-economically important nearshore fisheries and offer protection to coastal economies from extreme storm events [165–168].

The significance of mangrove ecosystem services has been recognised in Vietnam since the 1970s. Starting in 1978 and continuing to the present-day, numerous State-funded and NGO-supported projects have been undertaken [169, 170]. This has resulted in *c*. 43,750 ha of restored mangrove. However, despite recognised successes in reducing the rate of mangrove loss [171] nationally over the period 1983–2013, only the coastline from the Do Son Cape to Lach Truong River in RRD has seen a modest net gain (+7127 ha) in mangrove forest area [172]; a zone including Ninh Binh Province [173].

Since 2007, the Vietnamese Government has legislated a further nine key polices, strengthening the legal framework for mangrove conservation and increasing available funding [174, 175]. Challenges remain, however, such as navigating the relationship between infrastructure expansion and conservation; provisions for enforcement; addressing a sometimes-disproportionate focus towards supporting new forest plantations over the protection of existing ones; robust monitoring and evaluation systems; and equitable benefit-sharing to incentivise local community involvement [172, 175]. These matters are compounded at the most basic level by a lack of agreement over the reported extent of mangrove forestation. In 1998, Blasco et al. [176] pointed out that global records of what constitutes mangrove coverage could include a range of plant community-types and even areas of former mangrove converted to other uses; an inconsistency that problematised assessment and valuation. This continues to be the case. Estimates of the extant coverage in Vietnam 2000–2014 are illustrative and non-sequential: e.g., (2000) 210,000 ha [177], (2007) 168,689 ha [171], (2012) 254,000 [178], (2014) 157,500 ha [179].

The direct cost of mangrove restoration depends on the level of intervention required (e.g., from the cessation of logging to hydrological reconfiguration and hand planting). Estimates (excluding land purchase) range widely in accordance, from 225 US$/ha to over 200,000 US$/ha [180]. For Vietnam specifically, IUCN quotes a figure of 400–800 US$/ha for internationally supported reforestation projects [181]. Meta-analysis of available mangrove economic valuation literature for Southeast Asia produced an estimated mean value of 4185 US$/ha/year for the region’s mangrove ecosystem services, as of 2012 (excluding carbon sequestration, biodiversity, and recreational services) [178]. A detailed cost-benefit analysis of mangrove conservation and restoration for Ca Mau Province in southern Vietnam–including an estimate of carbon sequestration but excluding (among other values) recreation and biodiversity–calculated net benefits per ha for the year 2010 to be 1692.5 US$ [182]. On the basis of this latter snapshot calculation, if the country as a whole were to pursue a restorative baseline of *c*. 408,500 ha reported in 1943 (excluding back mangrove) [172, 183, 184], net benefit (unadjusted for inflation over the number of years required to reach such a baseline) could reach upwards of 700 million US$/ha/year; a substantial boost to local coastal economies.

While it is widely accepted that historical processes and changing coastal dynamics are important to the development of mangrove management and restoration [181, 185–187], and the relevance of palaeoecological data has been flagged [188, 189], with few exceptions [190–192], application to coastal or estuarine ecosystem restoration has lagged behind research in lacustrine and terrestrial environments [192]. Here we reference published results from Tràng An [109] to illustrate the role that palaeoecological data can play in such programmes, particularly with respect to site selection criteria and baseline mangrove community composition. Our findings show the importance of sub-coastal karst locations to the reestablishment of viable mangrove reserves and how palaeo-data permits restorative actions to be pursued pro-actively in protected locations where they might not otherwise be considered; an asset that enhances the existing positive gains and climate-change related benefits from forest restoration in an area of Vietnam’s coastline that is particularly vulnerable to erosion, storm, and inundation risks [193, 194].

**Fig 7:**
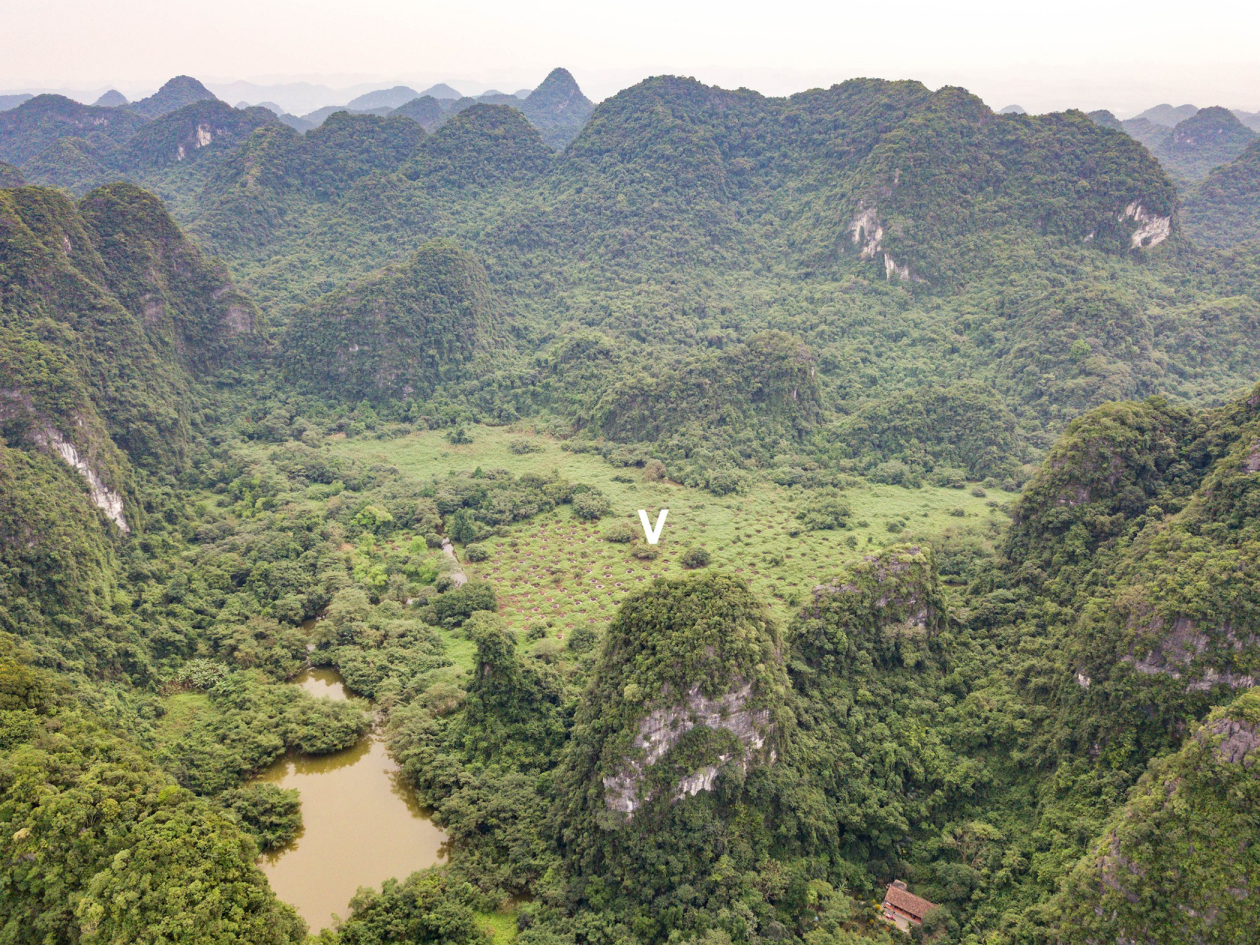
The Vung Tham doline in the centre of the Tràng An massif (2017). The white ‘V’ in the centre of the frame denotes the location of the coring site (105.89745°E, 20.25281°N) (Photo: Thorsten Kahlert).

Palaeoenvironmental reconstruction of Tràng An’s past vegetation (*see* Methods 2) has established the presence of mangrove habitats within a *c*. 20 ha doline in the interior of the massif (Fig 7) from 8100 cal. BP, and its persistence there until as recently as 300 years ago–long after the sea had regressed to modern levels from the Mid-Holocene high stand [109]. This late survival in the fossil record comprises taxa that are today found in back mangrove habitats requiring input of freshwater (such as, *Sonneratia*, *Excoecaria* and *Aegiceras* [109, 158, 195]), making these prime candidates to incorporate into preparatory measures that will support expansion of functional mangrove ecosystems [140]. *Sonneratia caseolaris* is already commonly used in mangrove restoration in northern Vietnam [196]; however, the palaeoecological data suggest that additional mangrove taxa that thrived here–including *Excoecaria agallocha*, *Aegiceras corniculatum*, *Bruguiera gymnorhiza*, *Xylocarpus granatum* and *Lumnitzera racemosa*–might be more appropriate choices for Tràng An and similarly situated inland sites currently, rather than species, such as *Rhizophora apiculata* and *Kandelia candel*, which are more commonly-used at seaward-facing restorative sites [170, 181, 196, 197].

While the socio-economic importance of mangrove restoration and governance is well-reported [166, 185, 193, 198], greater buy-in from all stakeholders, including local communities for whom clear identification and communication of benefits, beneficiaries, and potential risks, involvement in decision-making and subsequent actions will be critical [199, 200]. Sediment deficits imposed by the damming, re-direction and channelisation of rivers [201], increased reporting on adaptive actions [202], and the enhancement of existing capacity among local stakeholder authorities to systematically define mangrove areas, as well as monitoring and reporting on the progress of forest reestablishment are among the additional challenges still to be met.

### Mammal defaunation and reintroduction

The impact of defaunation on tropical plant communities, through vertebrate range reduction, population decline, and local (or wider) extinction, is likely to be far-reaching [203], particularly with the majority of tropical woody plant species (50–75%, and potentially as high as 90%) dispersed by birds or mammals [204–206]. New evidence also suggests that defaunation compromises the ability of global plant communities to track climate change [207]. Given projected impacts, such interconnectivity underscores the urgency with which faunal and floral species conservation measures need to be pursued in tandem.

The restoration of biotic connectivity features in the Global Standard for Nature-based Solutions (Criterion 3, Indicator 3.4) [208]. However, research into the impact of defaunation on the structure and integrity of tropical forests remains in its early stages. To date, most studies continue to be based on realistic and precise population models of individual tree species, but realistic and generalised models that can better account for complex interactions within forest systems [209], and the extended time periods over which impacts can manifest [210, 211] are yet to emerge. While the potential impacts of reduced seed dispersal discussed here are widely considered, the impact of changes to seed predation–be it reduced by the loss of large vertebrates or compensated for by increases in other seed predator guilds such as insects and fungi–has still to be comprehensively assessed or integrated into predictive models [209, 212]. Observations and recommendations that can be made on the basis of the palaeoecological and zooarchaeological records highlight the relevance of deep-time evidence to this emerging field [213] and present potential avenues where these data can help efforts to address defaunation.

Southeast Asia’s limestone karst environments currently represent some of region’s most critically at-risk biodiversity ‘hotspots’ [214–222]. Across Vietnam, karst systems make up 18 percent of the country’s geography [223, 224], including three of its inscribed World Heritage sites–Ha Long Bay (1994), Phong Nha–Ke Bang (2003) and Tràng An (2014). With combined visitor numbers to Ha Long Bay and Tràng An in 2018 exceeding 10 million [225], even in the post-Pandemic era these protected reserves will likely remain essential to the country’s burgeoning ecotourism sector, wider future economic buoyancy [225, 226] and conservation initiatives.

At a regional scale, the precarious state of mammal biodiversity in karst zones is accentuated when it is compared against the biodiversity evidenced in the zooarchaeological record [159, 227, 228]. This trend is confirmed by archaeological excavations in Tràng An, where analysis of prehistoric vertebrate fauna has identified the presence of at least 19 genera of mammals ≥ 2 kg (18,700–11,200 cal. BP). This compares starkly to as few as six extant in the landscape today (Table 1, *see* Methods 3 & 4). Loss on this scale is liable to compromise biological networks [221–224], including the capacity for seed dispersal, especially among large-fruited or large-seeded plant species that occur in Tràng An–such as, *Dillenia indica* L. (Dilleniaceae; ‘Elephant apple’), *Artocarpus* spp. (Moraceae; congenerics of jackfruit, ‘chempedak’ and breadfruit), *Mangifera* spp. (Anacardiaceae; ‘wild mangoes’), *Garcinia* spp. (Clusiaceae; congenerics of mangosteen) and *Diospyros* spp. (Ebenaceae; congenerics of persimmon) [109, 195, 229–232].

**Table 1:** Mammalian genera (≥ 2 kg) identified from archaeological investigations in Tràng An by the SUNDASIA project compared to those recorded in the same landscape today and their major ecological role(s) with associated references.

Archaeological (Genus)	Extant	Major ecological role(s)
*Manis*	*-*	predator of social insects [233]
*Macaca*	X	seed disperser [233]
*Trachypithecus*	(reintroduced)	seed predator [233]
*Arctonyx*	X	seasonal seed disperser, mesopredator [234, 235]
*Arctogalidia*	*-*	potential seed disperser [236, 237]
*Paradoxurus*	*-*	seed disperser, mesopredator [233]
*Panthera*	*-*	top predator [233]
*Prionailurus*	X	mesopredator [233]
*Ursus*	*-*	seed disperser [233]
*Tragulus*	*-*	seed disperser [233]
*Rusa*	*-*	seed disperser, browser [233]
*Axis (= Hyelaphus)*	*-*	seed disperser, grazer [238]
*Muntiacus*	*-*	seed disperser, predator, browser [233]
*Hydropotes*	*-*	seed disperser, browser [239]
*Bos*	*-*	browser [233]
*Capricornis*	*-*	browser [233]
*Sus*	X	seed predator, nest builder [233]
*Hystrix*	X	seed predator [233]
*Atherurus*	*-*	seed predator [233]

**Fig 8:**
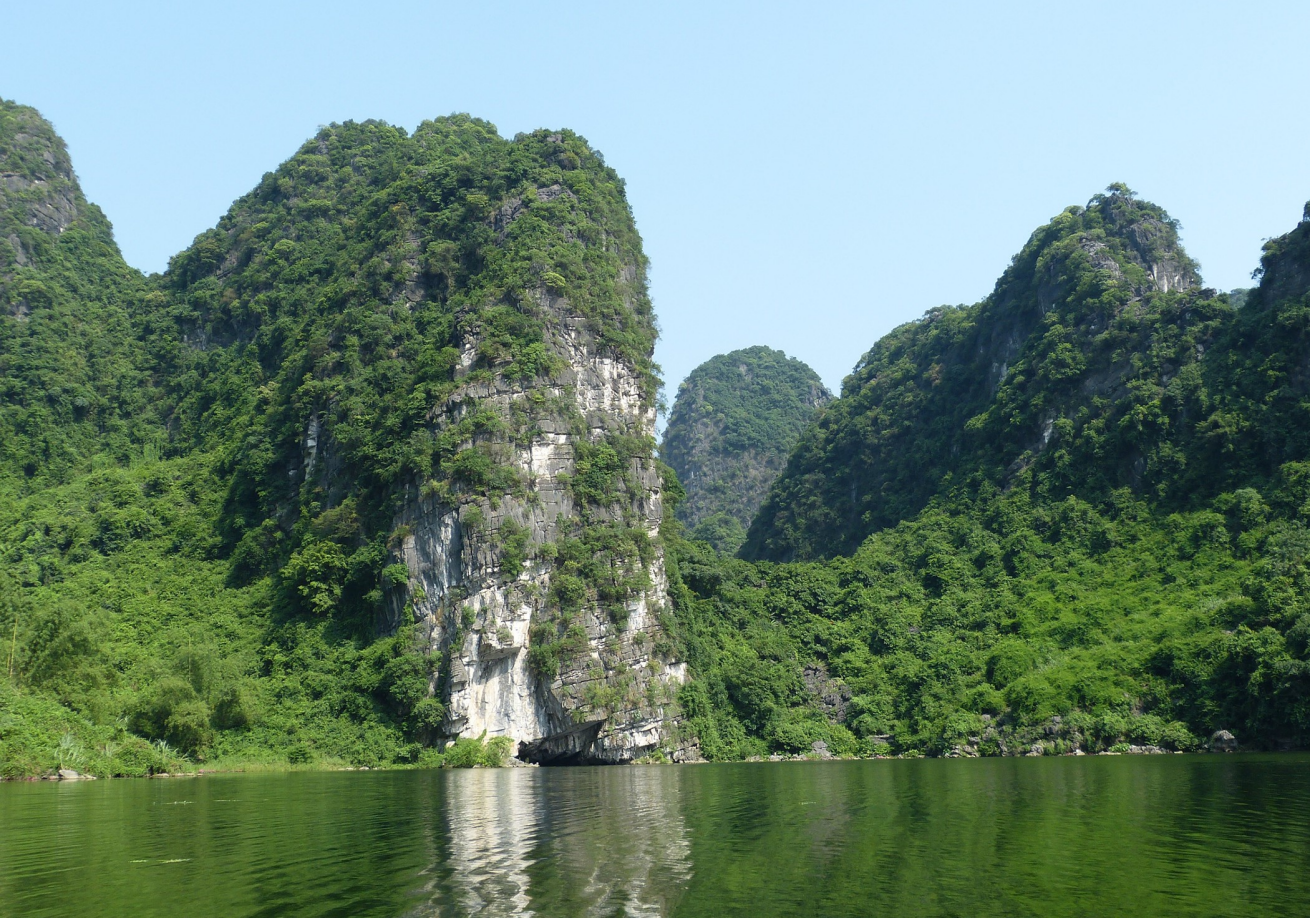
Vegetation cover on the Tràng An limestone massif. Palaeoenvironmental evidence from Tràng An indicates that broad habitat conditions found on the massif today extend back to at least the Late Pleistocene (Photo: Ryan Rabett).

The temporal persistence of biodiverse locales due to buffering influences such as topography and microclimatic diversity [240] is seen as an essential criterion to their functionality as reliable foci for conservation efforts [241], and for consideration as refugia against future climate change [242]. Palaeoenvironmental evidence from Tràng An has established that limestone forest vegetation prevalent here during the Late Pleistocene compares closely to that seen on the massif today [109, 243] (Fig 8). These findings suggest that the property’s complex topography has provided just such a buffering mechanism and raises its potential as an ecologically diverse setting capable of persisting through the impacts of future climate change. What is less clear is whether it will be able to do so in its currently defaunate state and, even if restoration measures can be instigated, if recovery will occur at a rate that will be quick enough to accommodate the pace of expected climate change, or where along that trajectory thresholds to (or away from) resilience and sustainability may be found.

In a region where ecosystem restoration is critically understudied [244], the multi-proxy evidence obtained from Tràng An for long-term habitat continuity, together with the archaeological recovery of key locally extinct vertebrate taxa demonstrably present in prehistory under similar conditions to those prevailing now [227, 245, 246], provides an opportunity to explore the risks and benefits of species reintroduction here.

Defined as *‘the intentional movement and release of an organism inside its indigenous range from which it has disappeared’* [228, 247: 3, 248, 249], reintroduction presents as a viable approach towards ‘rewilding’ an ecological system and towards bolstering self-regulation [250–252]. Although the concept of rewilding has itself generated considerable debate [253–255], the IUCN’s Commission on Ecosystem Management (CEM) Rewilding Thematic Group recently published (2021) a set of ten guiding principles to bring a more unified understanding of rewilding to the forefront of global conservation efforts [256]. Six of those principles relate directly to matters covered in this paper, including, the utilisation of wildlife; landscape scale planning; recognition of the dynamic nature of ecosystems; the use of reference ecosystems with relatively complete biota, degraded sites or historical data to compare the effects of reintroduction measures aiming to restore trophic interactions; the use of rewilding as a tool to mitigate climate change impacts; and engagement with local knowledge and stakeholders.

**Fig 9:**
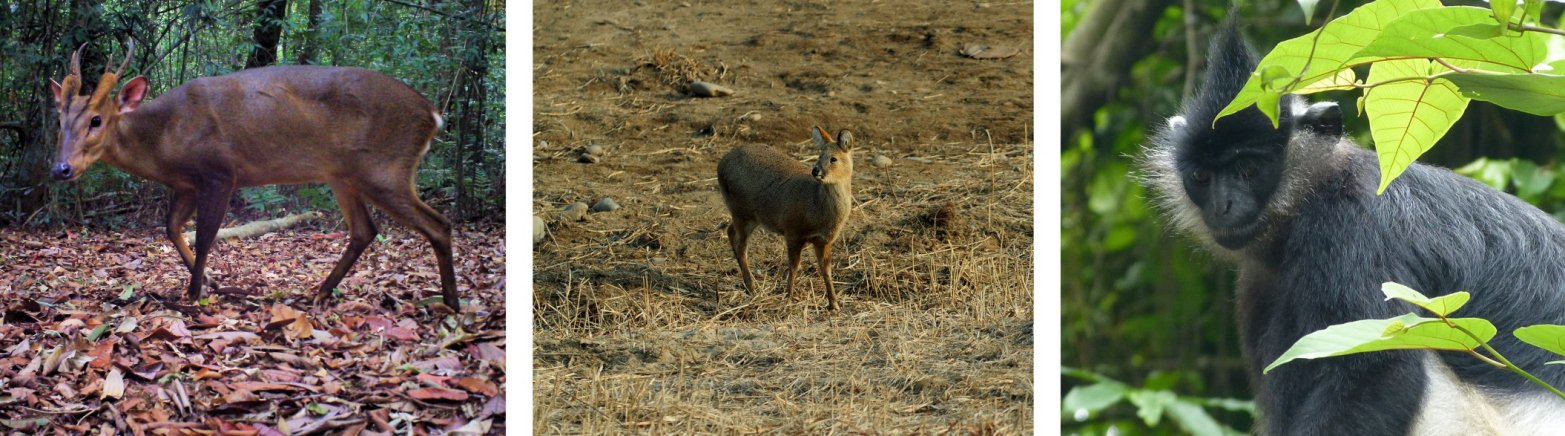
Potential candidates for refaunation in the Tràng An Landscape Complex World Heritage Site. Photo credits (left-right): Large-antlered muntjac (*Muntiacus vuquangensis*) (@Association Anoulak); Water deer (*Hydropotes inermis*) (Seong-Won Cho); Delacour’s langur (*Trachypithecus delacouri*) (Ryan Rabett).

In recognition of inherent uncertainties in seeking to re-establish complex extinct ecological networks, such principles need to be supported by science-based decision frameworks. These must systematically assess suitability between candidate species and potential release habitats, the ecological functions to be restored, and the range of likely costs and benefits of the action [249, 257]. Galetti et al. [251] have proposed that candidature may be favoured for species that ^1)^ have suitable existing stocks that can be drawn upon; ^2)^ are in the first instance lower tropic level generalists, which are suited to providing an ecological service that is impoverished or absent; ^3)^ do not represent a high health or economic risk to humans; ^4)^ can be managed in the event of increased abundance; and ^5)^ have comparatively small home ranges. We draw on these criteria with reference to three potential candidate species for reintroduction into Tràng An (Fig 9, Table 2). The case for the first two candidates is made on the basis of archaeological evidence; the third is based on the recent (1990s) presence here of the species in question and is already the subject of a trial reintroduction since 2020.

**Table 2:** Potential candidates for refaunation in the Tràng An Landscape Complex World Heritage Site. References pertaining to the large-antlered muntjac (*Muntiacus vuquangensis*) are extrapolated from congeneric data but are as yet unconfirmed for this species.

Refaunation candidate		Presence in Tràng An	IUCN Red List (global)	Feeding strategy	Home range & sociality	Economic/commercial impacts (local)	Ecological services/benefits	Failure risk
Large-antlered muntjac (*Muntiacus vuquangensis)*		Prehistoric^1^	Critically Endangered^2^	Terrestrial herbivore, concentrate selector frugivorous(?)^3,4^	Unknown *c*. 100 ha^5,6^ solitary	Unknown Potential forest edge crop raiding; population growth^2,7,8^	Unknown Potential dispersal agent of fruit-producing plants; seed predation; diverse range of plants in diet^4,9–13^	Snaring, habitat disturbance and loss, trade; poor management^2,13^
Water deer (*Hydropotes inermis*)		Prehistoric^14^	Vulnerable (decreasing) ^15^	Terrestrial herbivore, concentrate selector/intermediate (forbs & woody plants)^3,16,17^	$\bar{x}$ 277 ha^18^ solitary	Conflicting preference with humans for watered, open forest habitat–crop raiding; population growth^17,19,20^	Evidence emerging seed dispersal, (forbs notably), germination rate of dispersed seeds may decrease with gut passage (Korean data)^19,21^	Hunting, snaring, habitat loss, trade; poor management^15^
Delacour’s langur (*Trachypithecus delacouri*)		Historic^22^	Critically Endangered ^23^	Petrous folivore: young leaves 58%, mature leaves 20% (unripe fruit: 9–10%) of the diet^23,24^	20–50 ha^23^ gregarious	Population growth (long term)–e.g., quadrupled in 18yrs, Van Long Nature Reserve^25^	Unconfirmed seed predation; probable impact on plant growth/productivity (nutrient cycling), ecotourism^22,26^	Hunting, habitat loss, trade; poor management^23,25^
**Table No.**	**Reference name**	**Ref. No.**	**Table No.**	**Reference name**	**Ref No.**	**Table No.**	**Reference name**	**Ref No.**
1	Stimpson et al. (2019)	[245]	10	Chen et al. (2001)	[283]	19	Lee & Lee (2020)	[239]
2	Timmins et al. (2016)	[259]	11	Corlett (2007)	[233]	20	Chen et al. (2016)	[268]
3	Hoffman (1989)	[279]	12	Ilyas & Khan (2003)	[284]	21	Lee et al. (2021)	[287]
4	Sridhara et al. (2016)	[280]	13	Kumar et al. (2021)	[285]	22	Nadler (2015)	[288]
5	McCullough et al. (2000)	[260]	14	Stimpson et al. (2021)	[227]	23	Workman (2010)	[289]
6	Odden & Wegge (2007)	[261]	15	Harris & Duckworth (2015)	[270]	24	Workman & Schmitt (2012)	[290]
7	Steinmetz et al. (2010)	[258]	16	Kim et al. (2011)	[269]	25	Nguyen et al. (2019)	[291]
8	Thinley et al. (2017)	[281]	17	Schilling & Roessner (2017)	[273]	26	Berzaghi et al. (2018)	[204]
9	Brodie et al. (2009)	[282]	18	Kim & Lee (2011)	[286]			

The archaeological evidence stems from taxonomic identification of sub-fossil remains from extant solitary deer whose contribution to ecological processes has been underestimated [231, 258]. Both of the species in question are today considered to be internationally threatened and subject to range contraction. The Critically Endangered large-antlered muntjac, *Muntiacus vuquangensis* (syn. *M*. *Gigas*), is presently restricted to the Annamite (Truong Son) Mountains of Lao PDR, Vietnam, and eastern Cambodia [259]. The home range of this species, as with many other aspects of its behavioural ecology [259], is still poorly understood, but if that of other muntjac species can be taken as indicative [260, 261], it may be *c*. 100 ha (1 km^2^). The status of the species shows continuing decline to the point where it is threatened with extirpation due to large-scale illegal snaring [262, 263]. Thirty-eight *M*. *vuquangensis* were relocated ahead of flooding for the Nam Theun 2 Hydroelectric Project in northern Laos to habitat away from the site of the reservoir, though in the absence of post-release monitoring, their current status is unclear [264] and likely declining [259]. *M*. *vuquangensis* is also listed among 40 globally threatened species whose survival is likely to be assisted by upgrading of the Dong Chau-Khe Nuoc Trong Watershed Protection Forest in Quang Binh province to ‘Nature Reserve’ [265], but further targeted initiatives will be needed to ensure the continuation of the species [266].

Only a small number of confirmed sub-fossil records of *M*. *vuquangensis* are documented, all of them otherwise from China, indicating probable range contraction over time [267]. The recovery of a mandible fragment identified to this species from excavations at Hang Boi, a cave site in the central area of Tràng An, and tightly dated to 11,400–11,100 cal. BP, supports that contention [245]. Taken together with its early Holocene presence under comparable environmental conditions to those of today; its primary consumer status and solitary behaviour; probable small home range that could be accommodated within Tràng An’s protected 6226 ha property; and potential contribution to restoring degraded ecological services make the large-antlered muntjac an engaging possibility for reintroduction. Against this, stakeholders must consider the limited available knowledge about its behavioural ecology and challenges of acquiring suitable stock. However, if these constraints can be addressed, trial reintroduction into Tràng An as a discrete initiative or as part of a network of small ‘recovery zones’ in a similar vein to those that have been shown to enhance population growth for its congener the red muntjac *Muntiacus muntjac* [258] could be explored under strict conditions.

The extant native range of the second archaeologically identified species, the water deer, *Hydropotes inermis*, is now restricted to enclaves in the eastern sub-coastal areas of the Yangtze Basin, the Zhoushan Archipelago and, somewhat more abundantly in the Korean peninsula [268, 269]. The creation of new protected areas is a recommended conservation action for *H*. *inermis* [270], building on the well-established programme in China [268, 271, 272]. Fossil and sub-fossil evidence that can be attributed confidently to *H*. *inermis* is exceedingly rare [273]. This makes the recovery of remains from at least two individuals at the Thung Binh 1 archaeological site in Tràng An a significant discovery that confirms the presence of this species in Vietnam 16,000–13,000 cal. BP [227]. Given that any discussion to reintroduce the water deer into Vietnam would not at this point rest on grounds of restoration to a historic or Holocene range, and since limited attention has been paid to the species’ potential role in ecosystem services [239], thorough assessment of its suitability would be essential before reversable trials [274] could be considered.

With regard to both deer species, although palaeoenvironmental evidence affirms habitat continuity within the Tràng An massif, establishing archaeologically whether or not either continued to be present beyond the Mid-Holocene high stand should be a necessary priority for future research. Equally, in line with developing standards in rewilding, establishing a comprehensive restorative programme for Tràng An and similar locations will require an adaptive, regularly updatable management scheme that incorporates close monitoring of ecological conditions against reference data [275–277], and that takes pro-active measures to integrate local communities into the initiative [278]. Ensuring this and wider buy-in from corporate and policymaking stakeholders is heavily dependent on the opening of dialogue between parties with ordinarily separate agendas by establishing common goals and language [251]. In the final section of this paper, we trace stakeholder relationships that are under-writing the trial reintroduction into Tràng An of the IUCN Critically Endangered Delacour’s langur, *Trachypithecus delacouri*.

### Knowledge exchange: Trial reintroduction of Delacour’s langur

**Fig 10:**
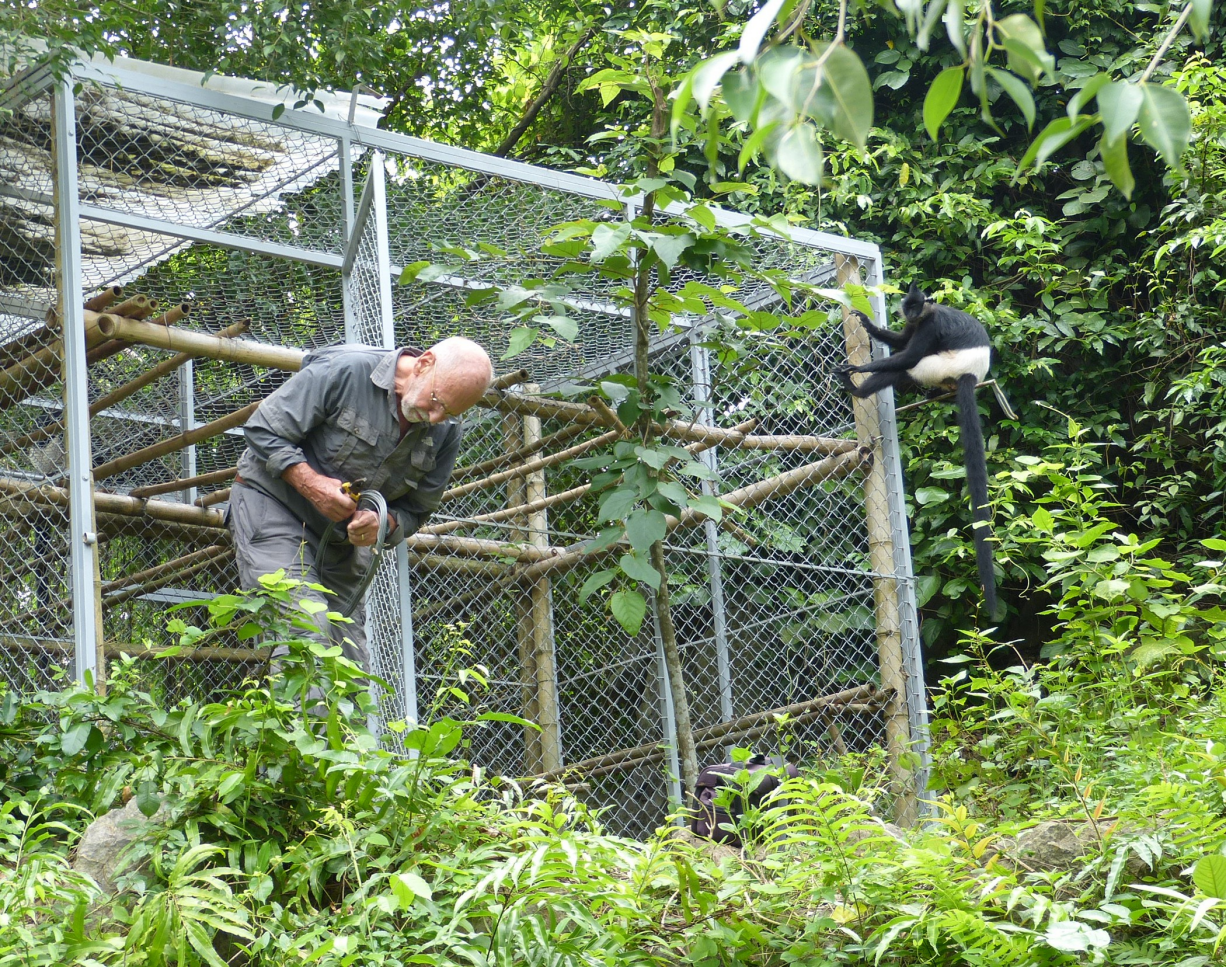
Monitoring visit to the Delacour’s langur trial reintroduction site on Ngoc Island, Tràng An (2022). The release cage was used for initial climatization purposes and continues to serve as contact location between the free-ranging primates and monitoring staff (Photo: Ryan Rabett).

The relationship between palaeo-research and its strategic utilisation emerged, in the context of Tràng An, through the practical demands of a World Heritage nomination. Over a period of two years (2012–14) this provided the stimulus for close collaboration between multiple independent stakeholders working at widely differing scales and in diverse knowledge domains. The dialogue and integration established during the nomination process has been preserved in the period since, for example in the formulation of management strategies and State of Conservation reports to (and monitoring by) the World Heritage Centre, and providing an additional steer to report requirements at local and national levels. Commitment from property management, provincial and national authorities, and corporate stakeholders to continue supporting scientific investigation has meant that channels of communication have remained highly active since the property’s 2014 inscription. This is exemplified through the Delacour’s langur reintroduction programme [292, 293] (Fig 10).

The potential to reintroduce the Delacour’s langur into Tràng An was identified in 2015 [287], and again in 2017, under Vietnam’s Prime Minister of Government Decision No: 628/QD-TTg [294]. When groundwork for trial reintroduction began in 2018, it drew together several separate research endeavours and professional fields. These included, ^1)^ a pre-existing arc of research work on this endemic Vietnamese species by the Endangered Primate Research Center and Cuc Phuong National Park; ^2)^ the opportune position of the SUNDASIA Project to promote high-level dialogue, field expertise, and immediate access to funds for training and outreach; ^3)^ willing managerial investment in administrative and legislative support through the Ninh Binh Department of Tourism and the Xuan Truong Construction Enterprise; and critically, ^4)^ the support and involvement of local communities. The procedural form that the trial has taken followed the ecotourism and local community-vested model that has proven so effective at building population numbers of this species in the nearby Van Long Nature Reserve [291, 295].

Recent genomic and ecological niche modelling analysis of the Françoisi Group of langurs, which includes *T*. *delacouri*, raises the possibility that the restricted distribution seen in surviving species of limestone langur to pockets of karst terrain may not, as previously postulated [289], be a consequence of historical human pressure but rather part of an ecological specialisation to this habitat [296, 297]. Thus, the Tràng An initiative marks a small triumph for the immediate survival of this species within its historic and potentially indigenous range; one reinforced by recent (Oct. 2021 & Sept. 2022) births of the first infants to the reintroduced troop. It expands opportunities to better understand the contribution of this species to karst forest ecosystem services [298]–particularly in a setting subject to sea-level change; and advances a positive relationship between biodiversity and tourism through a rewilding lens [253]. For the property, as a World Heritage Site, it also signals an evolution of management capacity, paving the way for creation of a formal conservation and adaptation strategy that will contribute to Government policy objectives, such as the Vietnam National Biodiversity Strategy to 2020 Vision to 2030, and international programmes, such as the Post-2020 Global Biodiversity Framework and UN Decade on Ecosystem Restoration. The Tràng An experience illustrates a simple fact: that the drive towards conserving global natural and cultural heritage, and the rehabilitation of environments in response to the current Climate and Biodiversity crises provides precisely the kind of forum where stakeholders from traditionally separate fields can find commonality of purpose and mutual benefit alongside collective responsibility.

## Discussion

Steffen et al. [64] make the case for active planetary stewardship to mitigate climate change. They propose that humanity be recast as an integral and integrating component of an adaptive Earth system rather than an external force driving change. The present generation of IAMs (such as DIVA), have enhanced our capacity to simulate real-world complexity, but have tended to retain a focus on least-cost conditions and aggregate global or regional scales. More empirical data is needed urgently to ground truth such predictions. This is recognised as an essential next step in the development of adaptive options that adequately account for local circumstances and variables [299, 300], the systemic impact of tipping-points [61, 63], and time-dependant changes to ecological services [211, 213]. Palaeoecological and archaeological records are uniquely placed to help meet that need.

In the context of this paper, we recognise that there continues to be significant challenges to reconstructing past coastal change within active deltaic systems. Available datasets tend to be few in number, creating low spatial and temporal resolution for assessing complex processes. While the RRD is comparatively well-represented, with >20 sedimentary cores, most radiometrically dated [141], compared to, for example, *c*. 36 cores from the larger Mekong Delta [301], shifting distributary channels, differential influence from wave, tide, and fluvial processes, subsidence and uplift, long-shore current, and sea level fluctuations, and human activity affect morphological development [108, 302]. Limited standardisation between source data–including differing coordinate systems, project objectives, available measuring precision, and even agreement on what should constitute a representative coastline (e.g., between mean high-water [MHW], mean sea level [MSL] and mean low-water level [MLW])–also continue to problematise reconstruction [106, 303, 304].

These constraints inevitably place confidence limits on palaeo-coastline models [83]; however, it does not negate their relevance. Evidence of deep time change in deltaic environments can provide valuable reference conditions against which inundation risk can be measured [70], including ecosystem time-lag and other coping and response mechanisms that play out over timescales that may otherwise be overlooked by predictive models built exclusively on Late Holocene evidence [69, 71, 72]. For a location like Tràng An, which is predicted to become coastal by 2150 under all of the emission scenarios presented here, evidence that the massif was isolated during the Mid-Holocene transgression bears directly on long-term risk management planning; the opening of new research avenues in heritage conservation; and on the raising of visitor, community, and administrative awareness. All of which are reflected in the property’s palaeo-data informed World Heritage Management Plan for the period 2021–2025 (Vision to 2045).

The potential re-establishment of mangroves and the restoration of the Tràng An’s vertebrate community provide further specific local-scale examples of how reference to the past can aid efforts to mitigate climate change impacts and foster economic growth through ecotourism and conservation-focused habitat management. Such initiatives align with Criterion 4 (economic viability) of the Global Standard for Nature-based Solutions [208] and are in close accordance with advocation for ‘Ecosystem-based Adaptation’ (EbA) [305–307]. Impediments to widespread adoption of EbAs have included concern among policymakers about the delay between investment and returns (e.g., growth period for mangrove restoration); response efficacy–as severe climate events might overwhelm natural solutions; and a lack of consensus over how to measure effectiveness and standardise monitoring. While these issues are still to be addressed comprehensively, the benefits are now widely seen to outweigh the costs [200, 208, 306, 308].

Uncertainty exists about how defaunate or ‘empty’ forests in the tropics, now rapidly increasing in number [262, 309–311], will cope with this century’s climate upheavals [312]. The reintroduction of locally extinct species to restore ecological processes by rewinding back to a ‘baseline’ of forest stability has attracted considerable attention [211, 250]. Such baselines usually refer to an historical period when the environmental effects of modern encroachment are considered to have been minimal [250, 313]. The scale of historically recent impacts to Southeast Asian biodiversity has been undoubtedly grave [314]; however, with few exceptions, studies are restricted to a time-depth that is rarely deeper the 19^th^ Century. Sophisticated ecological models are now able to help identify deep-time refugia, extending back thousands of years, providing supportive evidence for future conservation priorities [315]; however, neither line of research gives consideration to the presence or consequence of early human activity, which lies at the root of any recasting of our role within an integrated Earth system.

Over the last two decades, archaeological and palaeoecological research in South and Southeast Asia has demonstrated that tropical forests were not in a ‘pristine’ state prior to historical incursions [14, 23, 24, 26, 274, 316–318]. A detailed picture of the scale and nature of impacts due to low-level cumulative human activity over tens of thousands of years is only now starting to be documented [109, 227, 245, 309, 319–321]. However, the body of evidence that early humans were active agents in these environments is now such that the suitability–and by extension, presumed greater sustainability–of baselines drawn immediately prior to colonial periods is open to question. To be clear, human impact on tropical and other environments during prehistory was at a very low level compared to the far-reaching effects of global industrialisation over the last two hundred years [322], but it continued over much larger timescales. As such, to discount the presence and influence of prehistoric humanity is to introduce an unquantified bias into our picture of how modern environments have developed [16]. Archaeology and palaeoecology are key to addressing this imbalance by contributing site-based (i.e., local) data that enhance our understanding of extinct ecological networks and restorative measures. In this, the work in Tràng An is illustrative.

A significant challenge to opening pathways from the past to help address issues of the present day, remains the effectiveness of knowledge transfer. The importance of drawing together scientific results from across disciplines to inform and regulate international policy is widely recognised [323, 324]. When this process has been closely assessed though, such as through the RIU (Research, Integration, Utilisation) model [28, 29], the effectiveness of that transferability is constrained by independent agendas and motivations [325, 326]. Establishing a common conceptual framework is likely to be essential for an integrated response to the kinds of global issues now faced and the local specifics of how to manage them. While the approach described in this paper is context-specific to primarily Vietnamese, UK, and EU stakeholders, it serves as proof that partners with differing agendas, power, specialism and even language can align sufficiently to achieve this goal.

## Conclusion

In this paper we have provided empirically based examples of the contributions that palaeo-data can make to next generation IAMs that require enhanced provision for local and pro-active adaptations [300]. The detailed Digital Elevation Model (DEM) (*see* Methods 1) and sea level history [100] that now exist for the Tràng An World Heritage Site contribute to the growing body of past sea level data now available for the RRD and regionally, and locally specific insights into the effects of predicted inundation. We underscored the relevance to future coastal change scenarios through comparison to past coastal configurations and through identification of eight modern foci (coastal change analysis, hydrometeorological risk, economic adaptation, ocean-climate systems and feedback, coastal stabilisation, ecosystem restoration, sediment transport and salinity intrusion) that can draw directly from the Holocene evolution of the RRD. In this respect, the Tràng An World Heritage Site provides an excellent ‘anchor-point’ [100] of the sort that will need to be incorporated into new coastal vulnerability models to enhance their spatial-temporal resolution.

We have demonstrated how the persistence of back mangrove elements in the property’s interior until 300 years ago offers proof of habitat continuity and thus investment value to its restoration. Arguably, the re-establishment of mangroves in this and similar locations has the potential to provide a pre-emptive aid to mitigating sea level rise and to future-proof economic growth profiles. We further show through palaeoenvironmental evidence that the limestone forest vegetation prevalent in Tràng An during the Late Pleistocene compares closely to that seen on the massif today [109, 243]. This suggests that the property’s complex topography has long-provided climate-buffering characteristics, including during the Holocene inundation of the RRD. This fact signals its potential to do so again under projected climate change. Tempering this, however, our zooarchaeological findings have revealed the extent of the habitat’s defaunate state, and the importance of efforts to strengthen essential ecosystem services that will help ensure future habitat resilience. The rate of ecological service recovery required to avert triggering critical thresholds along the current trajectory of anthropogenic climate change will, however, require further research.

Finally, we have sought to demonstrate how simulation scenarios that do not consider the perspective afforded by archaeological and palaeoecological research risk missing the impacts of long-term feedback mechanisms and of creating misaligned or even maladaptive responses to climate change. The challenge facing archaeologists and palaeoecologists is to achieve greater integration and knowledge transfer of their research into policy-driving models. This will require flexibility in viewpoint on their part, and the identification of research pathways that are sensitive to and can dovetail with mainstream debates (without being run by them) on the convergence of climate and economics, and the IAM-based simulations that remain central to predicting their near-term course. Such actions will only be successful though if other stakeholders, including other scientific disciplines, and local partners are receptive to the contribution that prehistoric science can make. The Tràng An collaborative model provides one example of how these objectives can be realised.

## Methods

### 1. Comparison between RRD time-series and Climate Central coastline models

The Tràng An DEM was generated from three sources: existing digital elevation data supplied by the Vietnam Institute of Geosciences and Mineral Resources obtained during the property’s World Heritage nomination; available LiDAR data (0.5 m/pix) covered 29.19% of southern and eastern portions of the core zone (1797/6156 ha); and an unmanned aerial vehicle (UAV) survey programme, covering a further 24.5% of the core zone, giving a total coverage of 3315/6156 ha (53.75%). The UAV survey took place over the course of four field seasons using a small DJI MAVIC Pro and the Structure from Motion software. A total of 73 flights (excluding repeats) were flown at altitudes ranging 160–300 m. From this was produced 39 overlapping sectors, each 35 ha. Local Real-Time Kinematic (RTK) network access, retrieved using a Leica GS15 GNSS (Global Navigation Satellite System) nRTK (network Real Time Kinematic) unit, established x,y,z coordinates of each geographic position in real time to centimetre precision. The DroneDeploy app for iOS and its web equivalent were used to plan and conduct each survey sector as an autonomous flight. Collectively, these data provided a high resolution (0.14 m) digital surface model of a central corridor (2 x 7 km) across the Tràng An massif, covering an area that contained focal points of research (e.g., excavations, sampling and core collection sites) during the SUNDASIA Project. Onto this DEM we mapped the location and elevation of 27 corrosion notches using the Leica GS15 nRTK receiver and a Leica TS06 total station–see Kahlert et al. [100]–and later, a Leica BLK360 imaging laser.

Future coastline models were calculated from Climate Central’s re-worked Shuttle Radar Topography Mapping Digital Surface Model (SRTM DSM) [101, 102] using projected sea levels available through the NASA IPCC AR6 Sea Level Projection Tool (SLPT) [103–105] that are localised to Hon Dau (https://sealevel.nasa.gov/data_tools/17). Three temporal horizons (2050, 2100 & 2150) were considered in reference to the SLPT under three principal models, providing a less likely but more extreme scenario with medium and low confidence (SSP5–8.5), and a more likely scenario (SSP2–4.5 medium confidence). The medium confidence scenarios exclude processes (such as ice sheet development) that are not well understood. Using a raster calculator, values below the cut off point for each scenario were selected and converted into vector layers. Areas that were not connected to open water were eliminated from the coastline models, leaving only areas inundated that are directly connected to the sea or indirectly connected to it via a river. The IPCC predictions were overlain with palaeo-coastline reconstructions from the Tràng An project data [100] and available literature [96, 108, 141–145, 327]. Overlaying of available palaeo-coastlines and SLPT data layers was guided by matching modelled future submerged land (https://www.climatecentral.org/) and past sea levels relative to land elevation changes analogous to the IPCC AR6 predictions.

### 2. Palaeoenvironmental reconstruction

Comparative data on the modern floristic structure, composition and taphonomic pathways was obtained through the setting of 24 pollen traps in late 2017. These were positioned at open-air and cave settings to catch the pollen rain from vegetation in the vicinity of sites from where sub-fossil pollen assemblages were extracted to aid identification and help clarify and quantify taphonomic relationships between the outflow of pollen rain and levels of incorporation into sedimentary deposits. Surviving traps (*n* = 13) were collected for analysis in late 2018. Additionally, and dictated by terrain accessibility, an herbarium of fertile plants (*n* = 76 genera) was collected from primarily valley bottom habitats, many of which are currently characterised by disturbed, edge-adapted and re-growth communities. Duplicate collections were lodged with the Academy of Science and Technology, Hanoi; Forest Inventory & Planning Institute, Hanoi; Hanoi College of Pharmacy, and the Herbarium at the Royal Botanic Gardens, Kew.

These data informed a palaeoenvironmental assessment of vegetation conditions in Tràng An [195]. Sediments were analysed from two archaeological cave sites of differing elevation and aspect in order to assess any divergence in the accumulation of palaeo-vegetation proxies and to improve the assessment of preservation and inter-site comparison. Five cubic centimetres of sediment were volumetrically sub-sampled from 16 samples. Pollen extraction followed [328], excluding acetolysis as marginal preservation was anticipated. In the landscape, following exploratory augering at five promising sites, one manual sediment core (3.82 m), using a modified Livingston corer (Vung Tham) and two mechanised cores (from the dolines of Thung Ui, 13 m and Vung Chay, 9.91 m) were extracted with the assistance of the Tràng An Management Board and the Xuan Truong Construction Enterprise. Preliminary physical description of all cores was carried out in the field at the point of extraction. Core sections were kept in cold storage ahead of shipment under formal agreement and license to Queen’s University Belfast. Analytical measures applied in the laboratory analysis of the Vung Tham core, discussed in this paper, included magnetic susceptibility (measured at 2 cm increments), and 38 paired sub-samples taken at 10 cm intervals for loss-on-ignition and parallel pollen analysis. The Vung Tham stratigraphic sequence is anchored against six AMS radiocarbon dates spanning the period 8177–8026 cal. BP to 189–142 cal. BP (at 2 sigma). Full details of field and laboratory procedures relating to recovery of the Vung Tham core are presented in O’Donnell et al. [109].

### 3. Sub-fossil vertebrate fauna analysis

Faunal remains were principally recovered at the point of excavation or during trench-side sieving (2 mm gauge dry sieve) or during the processing of bulk sediment samples subsequently. All material was catalogued to stratigraphic context and inventoried in the field before being shipped back to the UK under formal authorization and quarantined before study. Identification to element and taxon was carried out through comparative analysis to the extensive skeletonised collections at the Oxford University Museum of Natural History, supplemented by reference to materials held at the University of Cambridge Museum of Zoology, Natural History Museum, London, and American Museum of Natural History, New York. In addition to morphological comparison, statistical assessment of dental metrics was carried out on samples of a range of cervid species to test for difference from normative values for each candidate taxon. Full analytical details relating to *Muntiacus vuquangensis* (syn. *M*. *Gigas*) and *Hydropotes inermis* are presented in Stimpson et al. [227, 245].

### 4. Trail camera survey

A pilot study of extant medium-large fauna in the core zone of Tràng An was undertaken using motion-activated, static 24MP trail cameras with infrared flash (Bushnell Trophy Cam Aggressor HD No Glow 24MP Camo). Seven units were deployed in accordance with protocols for the passive monitoring of mammals in the forested tropics [329]. The survey window was from September 2017 to April 2018, producing 1013 camera-trapping days (days x nos. camera units deployed) of footage from an area of 3 km^2^. Triggers recording wild animal activity were rare (*n* = 7/114, 6.1% events–where an ‘event’ represents a series of images pertaining to the same trigger). The remaining 107 events were triggered by the passage of people, domestic animals, or foliage movement [120, 195].

### 5. Ethics statement

The field research leading to the results discussed in this paper was undertaken at the written invitation of the Ninh Binh Provincial People’s Committee (Mr Tống Quang Thἰn, Chairman), 18 Nov. 2016 (document no. 253/UBND-VP9) and in accordance with subsequent provincial documents.: 31/UBND-VP9, 37/UBND-VP9, 174/UBND-VP9, and 206/UBND-VP9. The project was approved by the Ministry of Culture, Sports & Tourism document no. 2948/QI-)-BVHTTDL, signed by Đặng Thị Bích Liên—Deputy Minister, on 31 Aug. 2016.

## References

[1] ICOMOS Climate Change and Cultural Heritage Working Group. The Future of Our Pasts: Engaging Cultural Heritage in Climate Action. Paris: ICOMOS; 2019. Available from: https://www.icomos.org/en/77-articles-en-francais/59522-icomos-releases-future-of-our-pasts-report-to-increase-engagement-of-cultural-heritage-in-climate-action

[2] IPCC Climate Change 2001: Impacts, Adaptation, and Vulnerability. Contribution of the Working Group II to the Third Assessment Report of the Intergovernmental Panel on Climate Change. Cambridge: Cambridge University Press; 2001. Available from: https://www.ipcc.ch/site/assets/uploads/2018/03/WGII_TAR_full_report-2.pdf

[3] IPCC Climate Change 2007: Impacts, Adaptation and Vulnerability. Contribution of Working Group II to the Fourth Assessment Report of the Intergovernmental Panel on Climate Change. Cambridge: Cambridge University Press; 2007a. Available from: https://www.ipcc.ch/site/assets/uploads/2018/03/ar4_wg2_full_report.pdf

[4] IPCC Climate change: The Physical Science Basis (Summary for policymakers). Contribution of Working Group 1 to the Fourth Assessment Report of the Intergovernmental Panel on Climate Change. Cambridge: Cambridge University Press; 2007b. Available from: https://www.ipcc.ch/report/ar4/wg1/

[5] IPCC Managing the Risks of Extreme Events and Disasters to Advance Climate Change Adaptation. A Special Report of Working Groups I and II of the Intergovernmental Panel on Climate Change. Cambridge: Cambridge University Press; 2012. Available from: https://www.ipcc.ch/report/managing-the-risks-of-extreme-events-and-disasters-to-advance-climate-change-adaptation/

[6] IPCC Climate Change 2014: Impacts, Adaptation, and Vulnerability. Part A: Global and Sectoral Aspects. Contribution of Working Group II to the Fifth Assessment Report of the Intergovernmental Panel on Climate Change. Cambridge: Cambridge University Press; 2014. Available from: https://www.ipcc.ch/report/ar5/wg2/

[7] Markham A, Osipova E, Lafrenz Samuels K, Caldas A. World Heritage and Tourism in a Changing Climate. Nairobi, Kenya: UNEP and UNESCO; 2016. Available from: https://whc.unesco.org/en/tourism-climate-change/

[8] Gills B. & Morgan J. Global Climate Emergency: after COP24, climate science, urgency, and the threat to humanity. Globalizations. 2020;17(6): 885–902. doi: 10.1080/14747731.2019.1669915

[9] Seddon AWR. Special feature: Measuring components of ecological resilience in long-term ecological datasets. Biol Letters. 2021;17: 20200881. doi: 10.1098/rsbl.2020.0881 33497590 PMC7876599

[10] Hanley N, Tinch D, Angelopoulos K, Davis A, Barbier EB, Watson F. What drives long-run biodiversity change? New insights from combining economics, palaeoecology and environmental history. J Environ Econ Manag. 2009;57: 5–20. doi: 10.1016/j.jeem.2008.03.005

[11] Edwards TL, Crucifix M, Harrison SP. Using the past to constrain the future: how the palaeorecord can improve estimates of global warming. Progress Phys Geogr. 2007;31: 481–500. doi: 10.1177/0309133307083295

[12] Masson-Delmotte V, Schulz M, Abe-Ouchi A, Beer J, Ganopolski A, Rouco JFG, et al. Information from Paleoclimate Archives Ch5 Climate Change 2013: The Physical Science Basis. Contribution of Working Group I to the Fifth Assessment Report of the Intergovernmental Panel on Climate Change. Cambridge: University of Cambridge; 2013. Available from: https://www.ipcc.ch/site/assets/uploads/2018/02/WG1AR5_Chapter05_FINAL.pdf

[13] Lunt DJ, Elderfield H, Pancost R, Ridgwell A, Foster GL, Haywood J, et al. Warm climates of the past–a lesson for the future? Phil Trans R Soc A. 2013;371: 20130146. doi: 10.1098/rsta.2013.0146 24043873 PMC3785815

[14] Boivin N & Crowther A. Mobilizing the past to shape a better Anthropocene. Nat Ecol Evol. 2021;5: 273–284. doi: 10.1038/s41559-020-01361-4 33462488

[15] Boivin NL, Zeder MA, Fuller DQ, Crowther A, Larson G, Erlandson JM. Ecological consequences of human niche construction: Examining long-term anthropogenic shaping of global species distributions. Proc Natl Acad Sci USA. 2016;113(23): 6388–6396. doi: 10.1073/pnas.1525200113 27274046 PMC4988612

[16] Ellis EC, Kaplan JO, Fuller DQ, Vavrus S, Goldewijk KK, Verburg PH. Used planet: A global history. Proc Natl Acad Sci USA. 2013;110(20): 7978–7985. doi: 10.1073/pnas.1217241110 23630271 PMC3657770

[17] Hussain ST & Reide F. Paleoenvironmental humanities: Challenges and prospects of writing deep environmental histories. WIREs Climate Change. 2020;11: e667. doi: 10.1002/wcc.667

[18] Lane PJ. Archaeology in the age of the Anthropocene: A critical assessment of its scope and societal contributions. J Field Archaeol. 2015;40(5): 485–498. doi: 10.1179/2042458215Y.0000000022

[19] Nogues-Bravo D, Rodríguez-Sánchez F, Orsini L, de Boer E, Jansson R, Morlon H, et al. Cracking the code of biodiversity responses to past climate change. Trends Ecol Evol. 2018;33(10): 765–776. doi: 10.1016/j.tree.2018.07.005 30173951

[20] Pisor AC & Jones JH. Human adaptation to climate change: An introduction to the special issue. Am J Hum Biol. 2020; e23530. doi: 10.1002/ajhb.23530 33230887

[21] Redman CL & Kinzig AP. Resilience of past landscapes: resilience theory, society, and the *longue durée*. Conserv Ecol. 2003;7(1): 14. http://www.consecol.org/vol7/iss1/art14

[22] Rick T, Ontiveros MAC, Jerardino A, Mariotti A, Méndez C, Williams AN. Human-environmental interactions in Mediterranean climate regions from the Pleistocene to the Anthropocene. Anthropocene. 2020;31: 100253. doi: 10.1016/j.ancene.2020.100253

[23] Roberts P, Hunt C, Arroyo-Kalin M, Evans D, Boivin N. The deep human prehistory of global tropical forests and its relevance for modern conservation. Nature Plants. 2017;3: 17093. doi: 10.1038/nplants.2017.93 28770831

[24] Roberts P, Bovin N, Kaplan JO. Finding the Anthropocene in tropical forests. Anthropocene. 2018;23: 5–16. doi: 10.1016/j.ancene.2018.07.002

[25] Roscoe PA. Changing climate for anthropological and archaeological research? Improving the climate-change models. Am Anthropol. 2014;116(3): 535–548. doi: 10.1111/aman.12115

[26] Stephens L, Fuller D, Boivin N, Rick T, Gauthier N, Kay A, et al. Archaeological assessment reveals Earth’s early transformation through land use. Science. 2019; 365(6456): 897–902. doi: 10.1126/science.aax1192 31467217

[27] Burke A, Peros MC, Wren CD, Pausata FSR, Riel-Salvatore J, Moine O, et al. The archaeology of climate change: The case for cultural diversity. Proc Natl Acad Sci USA. 2021;118(30): e2108537118. doi: 10.1073/pnas.2108537118 34301875 PMC8325276

[28] Bröcher M. How does science-based policy advice matter in policy making? The RIU model as a framework for analyzing and explaining processes of scientific knowledge transfer. Forest Policy Econ. 2016;68: 65–72. doi: 10.1016/j.forpol.2016.04.001

[29] Bröcher M & Krott M. The RIU model as an analytical framework for scientific knowledge transfer: the case of the “decision support system forest and climate change”. Biodiv Conserv. 2014;23: 3641–3656. doi: 10.1007/s10531-014-0820-5

[30] Giliberto F. Heritage for Global Challenges. A Research Report by PRAXIS: Arts and Humanities for Global Development. Leeds: University of Leeds; 2021. Available from: https://changingthestory.leeds.ac.uk/wp-content/uploads/sites/110/2021/02/Heritage-for-Global-Challenges-Report-2021.pdf

[31] Hill AC. COVID’s lesson for climate research: Go local. Nature. 2021;595: 9. doi: 10.1038/d41586-021-01747-9 34188218

[32] Hope C, Anderson J, Wenman P. Policy analysis of the greenhouse effect: An application of the PAGE model. Energ Policy. 1993;21(3): 327–338. doi: 10.1016/0301-4215(93)90253-C

[33] Weyant J. Some contributions of Integrated Assessment Models of global climate change. Rev Env Econ Policy. 2017;11(1): 115–137. doi: 10.1093/reep/rew018

[34] Tol RSJ & Fankhauser S. On the representation of impact in integrated assessment models of climate change. Environ Model Assess. 1998;3: 63–74. doi: 10.1023/A:1019050503531

[35] Hope C. The marginal impact of CO2 from PAGE2002: An integrated assessment model incorporating the IPCC’s five reasons for concern. Integrat Assess. 2006;6(1): 19–56.

[36] Plambeck EL, Hope C, Anderson J. The PAGE95 model: integrating science and economics of global warming. Energ Econ. 1997;19(1): 77–101. doi: 10.1016/S0140-9883(96)01008-0

[37] Holman IP, Brown C, Carter TR, Harrison PA, Rounsevell R. Improving the representation of adaptation in climate change impact models. Reg Environ Change. 2019;19: 711–721. doi: 10.1007/s10113-018-1328-4 30956567 PMC6418063

[38] Tompkins EL & Eakin H. Managing private and public adaptation to climate change. Global Enviro Change. 2012;22(1): 3–11. doi: 10.1016/j.gloenvcha.2011.09.010

[39] Harrison PA, Dunford RW, Holman IP, Rounsevell MDA. Climate change impact modelling needs to include cross-sectoral interactions. Nat Clim Change. 2016;6(9): 885–890. doi: 10.1038/nclimate3039

[40] Fischer H, Meissner KJ, Mix AC, Abram NJ, Austermann J, Brovkin V, et al. Palaeoclimate constraints on the impact of 2°C anthropogenic warming and beyond. Nat Geosci. 2018;11: 474–485. doi: 10.1038/s41561-018-0146-0

[41] Valdes P. Built for stability. Nature Geoscience. 2011;4: 414–416. doi: 10.1038/ngeo1200

[42] Fisher-Vanden K, Wing IS, Lanzi E, Popp D. Modeling climate change adaptation: challenges, recent developments, and future directions. Boston University. 2011. Available from: http://people.bu.edu/isw/papers/impacts_adaptation_modeling.pdf

[43] Hyun JH, Kim JY, Park CY, Lee DK. Modeling decision-maker preferences for long-term climate adaptation planning using a pathways approach. Sci Total Environ. 2021;772: 145335. doi: 10.1016/j.scitotenv.2021.145335 33581530

[44] OECD The Economic Consequences of Climate Change. Paris: OECD Publishing; 2015. doi: 10.1787/9789264235410-en

[45] Asefi-Najafabady S, Villegas-Ortiz L, Morgan J. The failure of Integrated Assessment Models as a response to ‘climate emergency’ and ecological breakdown: The Emperor has no clothes. Globalizations. 2020. Pp. 11 doi: 10.1080/14747731.2020.1853958

[46] Keen S. The appallingly bad neoclassical economics of climate change. Globalizations. 2020. Pp. 29. doi: 10.1080/14747731.2020.1807856

[47] Palmer PI & Smith MJ. Model human adaptation to climate change. Nature. 2014;512: 365–366. doi: 10.1038/512365a 25164733

[48] Reilly J & Schimmelpfening D. Irreversibility, uncertainty, and learning: portraits of adaptation to long term climate change. Climatic Change. 2000;45: 253–278.

[49] Diaz DB & Moore F. Quantifying the economic risks of climate change. Nat Clim Change. 2017;7: 774–782. doi: 10.1038/NCLIMATE3411

[50] Moore FC, Rising J, Lollo N, Springer C, Vasquez V, Dulginow A, et al. Mimi-PAGE, an open-source implementation of the PAGE09 integrated assessment model. Sci Data. 2018;5: 180187. doi: 10.1038/sdata.2018.187 30251994 PMC6154285

[51] Mathias JD, Debeljak M, Deffuant G, Diemer A, Dierickx F, Donges JF, et al. Grounding Social Foundations for Integrated Assessment Models of Climate Change. Earth’s Future. 2020;8: e2020EF001573. doi: 10.1029/2020EF001573

[52] Hinkel J. DIVA: An Iterative Method for Building Modular Integrated Models. Adv Geosci. 2005;4. doi: 10.5194/adgeo-4-45-2005

[53] Brown S, Nicholls RJ, Lowe JA, Hinkel J. Spatial variations of sea-level rise and impacts: An application of DIVA. Climatic Change. 2016;134: 403–16. doi: 10.1007/s10584-013-0925-y

[54] Diaz DB. Estimating global damages from sea level rise with the Coastal Impact and Adaptation Model (CIAM). Climatic Change. 2016;137: 143–56. doi: 10.1007/s10584-016-1675-4

[55] Hinkel J, Nicholls R, Vafeidis A, Tol R, Avagianou T. Assessing risk of and adaptation to sea-level rise in the European Union: An application of DIVA. Mitig Adapt Strat Gl. 2010;15: 703–719. doi: 10.1007/s11027-010-9237-y

[56] Muis S, Verlaan M, Nicholls RJ, Brown S, Hinkel J, Lincke D, et al. A comparison of two global datasets of extreme sea levels and resulting flood exposure. Earth’s Future. 2017;5: 379–392. doi: 10.1002/2016EF000430

[57] Tamura M, Kumano N, Yotsukuri M, Yokoki H. Global assessment of the effectiveness of adaptation in coastal areas based on RCP/SSP scenarios. Climatic Change. 2019;152: 363–77. doi: 10.1007/s10584-018-2356-2

[58] Vafeidis AT, Nicholls RJ, McFadden L, Tol RSJ, Hinkel J, Spencer T, et al. A new global coastal database for impact and vulnerability analysis to sea-Level rise. *J* Coastal Res. 2008;24(4): 917–924. Available from: http://www.jstor.org/stable/40065185

[59] Wolff C, Vafeidis AT, Lincke D, Marasmi C, Hinkel J. Effects of scale and input data on assessing the future impacts of coastal flooding: An application of DIVA for the Emilia-Romagna coast. Front. Mar. Sci. 2016;3: 41. doi: 10.3389/fmars.2016.00041

[60] Wolff C, Vafeidis AT, Muis S, Lincke D, Satta A, Lionello P, et al. A Mediterranean coastal database for assessing the impacts of sea-level rise and associated hazards. Sci. 2018;5: 180044. doi: 10.1038/sdata.2018.44 29583140 PMC5870338

[61] Cai Y, Judd KL, Lenton TM, Lontzek TS, Narita D. Environmental tipping points significantly affect the cost−benefit assessment of climate policies. Proc Natl Acad Sci USA. 2015;112(15): 4606–4611. doi: 10.1073/pnas.1503890112 25825719 PMC4403162

[62] Grinsted A & Hesselbjerg Christensen J. The transient sensitivity of sea level rise. Ocean Science. 2021;17: 181–186. doi: 10.5194/os-17-181-2021

[63] Lenton TM, Rockström J, Gaffney O, Rahmstorf S, Richardson K, Steffen J, et al. Climate tipping points—too risky to bet against. Nature. 2019;575: 592–96. doi: 10.1038/d41586-019-03595-0 31776487

[64] Steffen W, Rockström J, Richardson K, Lenton TM, Folke C, Liverman D, et al. Trajectories of the Earth System in the Anthropocene. Proc Natl Acad Sci USA. 2018;115(33): 8252–59. doi: 10.1073/pnas.1810141115 30082409 PMC6099852

[65] Di Marco M, Harwood TD, Hoskins AJ, Ware C, Hill SLL, Ferrier S. Projecting impacts of global climate and land‐use scenarios on plant biodiversity using compositional‐turnover modelling. Global Change Biol. 2019;25: 2763–2778. doi: 10.1111/gcb.14663 31009149

[66] Meller L, van Vuuren DP, Cabeza M. Quantifying biodiversity impacts of climate change and bioenergy: the role of integrated global scenarios. Reg Environ Change. 2015;15: 961–971. doi: 10.1007/s10113-013-0504-9

[67] Verburg PH, Dearing JA, Dyke JG, van der Leeuw S, Seitzinger S, Steffen W, et al. Methods and approaches to modelling the Anthropocene. Global Enviro Change. 2016;39: 328–340. doi: 10.1016/j.gloenvcha.2015.08.007

[68] Barnett RL, Charman DJ, Johns C, Ward SL, Bevan A, Bradley SL, et al. Nonlinear landscape and cultural response to sea-level rise. Sci Adv. 2020;6: eabb6376. doi: 10.1126/sciadv.abb6376 33148641 PMC7673675

[69] Woodroffe CD & Murray-Wallace CV. Sea-level rise and coastal change: the past as a guide to the future. Quat Sci Rev. 2012; 54: 4–11. doi: 10.1016/j.quascirev.2012.05.009

[70] Helfensdorfer AM, Power HE, Hubble TCT. Modelling Holocene analogues of coastal plain estuaries reveals the magnitude of sea-level threat. Sci Rep. 2019;9: 2667. doi: 10.1038/s41598-019-39516-4 30804465 PMC6389980

[71] Pearson S, Lynch AJJ, Plant R, Cork S, Taffs K, Dodson J, et al. Increasing the understanding and use of natural archives of ecosystem services, resilience and thresholds to improve policy, science and practice. The Holocene. 2014;25(2):366–378. doi: 10.1177/0959683614558650

[72] Switzer AD, Sloss CR, Horton BP, Zong Y. Preparing for coastal change. Quat Sci Rev. 2012;54: 1–3. doi: 10.1016/j.quascirev.2012.09.005

[73] Nguyen HTT, Turner SWD, Buckly BM, Galelli S. Coherent Streamflow Variability in Monsoon Asia Over the Past Eight Centuries—Links to Oceanic Drivers. Water Resource Res. 2020;56: e2020WR027883. doi: 10.1029/2020WR027883

[74] Larsen F, Tran LV, Hoang HV, Tran LT, Christiansen AV, Pham NQ. Groundwater salinity influenced by Holocene seawater trapped in incised valleys in the Red River delta plain. Nat Geosci. 2017;10: 376–382. doi: 10.1038/NGEO2938

[75] Manzano S, Julier ACM, Dirk CJ, Razafimanantsoa AHI, Samuels I, Petersen H, et al. Using the past to manage the future: the role of palaeoecological and long-term data in ecological restoration. Restor Ecol. 2020;28(6): 1335–1342. doi: 10.1111/rec.13285

[76] Vacchi M, Pappalardo M. Sea level rise scenarios in a changing climate. Learning past to predict the future. Agrochimica–International Journal of Plant Chemistry, Soil Science and Plant Nutrition of the University of Pisa–Special Issue. Pisa University Press; 2019. Pp. 271–275.

[77] Hooijer A & Vernimmen R. Global LiDAR land elevation data reveal greatest sea-level rise vulnerability in the tropics. Nature Communications. 2021;12: 3592. doi: 10.1038/s41467-021-23810-9 34188026 PMC8242013

[78] Dasgupta S, Laplante B, Meisner C, Wheeler D, Yan J. The impact of sea level rise on Developing Countries: A comparative analysis. World Bank Policy Research Working Paper. 2007;4136.

[79] Eckstein D, Hutfils M-L, Winges M. Global Climate Risk Index 2019. Germanwatch. 2018. Available from: www.germanwatch.org/en/cri

[80] Neumann JE, Emanuel KA, Ravela S, Ludwig LC, Verly C. Risks of coastal storm surge and the effect of sea level rise in the Red River Delta, Vietnam. Sustainability. 2021;7: 6553–6572. doi: 10.3390/su7066553

[81] Woodroffe CD, Nicholls RJ, Saito Y, Chen Z, Goodbred SL. Landscape variability and the response of Asian megadeltas to environmental change. In: Harvey N, editor. Global Change and Integrated Coastal Management. Dordrecht: Springer; 2006. Pp. 277–314. doi: 10.1007/1-4020-3628-0_10

[82] Global Facility for Disaster Reduction & Recovery (GFDRR) Climate risk and adaptation: Country profile–Vietnam. World Bank Group. 2011.

[83] GFDRR. Country Profile: Vietnam. World Bank Group. 2015.

[84] Hens L, Thinh NA, Hanh TH, Cuong NS, Lan TD, Thanh NV et al. Sea-level rise and resilience in Vietnam and the Asia-Pacific: A synthesis. Vietnam J Earth Sci. 2018;40: 127–153. doi: 10.15625/0866-7187/40/2/11107

[85] Dougherty AJ, Thomas ZA, Fogwill C, Hogg A, Palmer J, Rainsley E, et al. Redating the earliest evidence of the mid-Holocene relative sea-level highstand in Australia and implications for global sea-level rise. PLoS ONE. 2019;14(7), e0218430. doi: 10.1371/journal.pone.0218430 31314758 PMC6636714

[86] Sweet W. V. & Park J. From the extreme to the mean: Acceleration and tipping points of coastal inundation from sea level rise. *Earth’s Future* 2, 579–600. 10.1002/2014EF000272 (2014).

[87] Lambeck K, Woodroffe CD, Antonioli F, Anzidei M, Gehrels WR, Laborel J, et al. Paleoenvironmental records, geophysical modeling, and reconstruction of sea-level trends and variability on centennial and longer timescales. In: Church JA, Woodworth P, Aarup T & Wilson W, editors. Understanding Sea-Level Rise and Variability. Oxford: Blackwell Publishing; 2010. Pp. 61–121. doi: 10.1002/9781444323276

[88] Mann T, Rovere A, Schöne T, Klicpera A, Stocchi P, Lukman M, et al. The magnitude of a mid-Holocene sea-level highstand in the Strait of Makassar. Geomorphology. 2016;257: 155–163. doi: 10.1016/j.geomorph.2015.12.023

[89] Majewski JM, Switzer AD, Meltzner AJ, Parham PR, Horton BP, Bradley SL, et al. Holocene relative sea-level records from coral microatolls in Western Borneo, South China Sea. The Holocene. 2018;28(9): 1431–1442. doi: 10.1177/0959683618777

[90] Mann T, Bender M, Lorscheid T, Stocchi P, Vacchi M, Switzer AD, et al. Holocene sea levels in Southeast Asia, Maldives, India and Sri Lanka: The SEAMIS database. Quat Sci Rev 2019;219: 112–25. doi: 10.1016/j.quascirev.2019.07.007

[91] Meltzner AJ, Switzer AD, Horton BP, Ashe E, Qiu Q, Hill DF, et al. Half-metre sea-level fluctuations on centennial timescales from mid-Holocene corals of Southeast Asia. Nat Commun. 2017;8: 14387. doi: 10.1038/ncomms14387 28186122 PMC5309900

[92] Boyd W & Lam D. Holocene elevated sea levels on the north coast of Vietnam. *Aust* Geogr Stud. 2004;42(1): 77–88. doi: 10.1111/j.1467-8470.2004.00244.x

[93] Hanebuth T, Stattegger K, Grootes PM. Rapid Flooding of the Sunda Shelf: A Late-Glacial Sea-Level Record. Science. 2000;288: 5468: 1033–1035. 10.1126/science.288.5468.1033 10807570

[94] Hanebuth TJJ, Stattegger K, Bojanowski A. Termination of the Last Glacial Maximum sea-level lowstand: The Sunda-Shelf data revisited. Glob Planet Change. 2009;66(1): 76–84. doi: 10.1016/j.gloplacha.2008.03.011

[95] Hanebuth T, Voris H, Yokoyama Y, Saito Y, Okuno JI. Formation and fate of sedimentary depocentres on Southeast Asia’s Sunda Shelf over the past sea-level cycle and biogeographic implications. Earth-Sci Rev. 2011;104: 92–110. doi: 10.1016/j.earscirev.2010.09.006

[96] Hori K, Tanabe S, Saito Y, Haruyama S, Nguyen V, Kitamura A. Delta initiation and Holocene sea-level change: example from the Song Hong (Red River) delta, Vietnam. Sediment Geol. 2004;164: 237–249. doi: 10.1016/j.sedgeo.2003.10.008

[97] Sathiamurthy E & Voris HK. Maps of Holocene sea level transgression and submerged lakes on the Sunda Shelf. Natur Hist J Chulalongkorn Univ (Supplement). 2006;2: 1–44.

[98] Tjia HD. Sea-level changes in the tectonically stable Malay-Thai Peninsula. Quatern Int. 1996;31: 95–101. doi: 10.1016/1040-6182(95)00025-E

[99] Yao Y, Harff J, Meyer M, Zhan W. Reconstruction of paleocoastlines for the northwestern South China Sea since the Last Glacial Maximum. Sci China D: Earth Sci 2009;52(8): 1127–1136. doi: 10.1007/s11430-009-0098-8

[100] Kahlert T, O’Donnell S, Stimpson C, Nguyen TMH, Hill. et al. Mid-Holocene coastline reconstruction from geomorphological sea level indicators in the Tràng An World Heritage Site, Northern Vietnam. Quaternary Sci Rev. 2021;236: 107001. doi: 10.1016/j.quascirev.2021.107001

[101] Kulp SA & Stauss BH. CoastalDEM: A global coastal digital elevation model improved from SRTM using a neural network. Remote Sens Environ. 2018;206: 231–239. doi: 10.1016/j.rse.2017.12.026

[102] Kulp SA & Stauss BH. New elevation data triple estimates of global vulnerability to sea-level rise and coastal flooding. Nat Commun. 2019;10: 4844. doi: 10.1038/s41467-019-12808-z 31664024 PMC6820795

[103] Fox-Kemper B, Hewitt HT, Xiao C, Aðalgeirsdóttir G, Drijfhout, Edwards TL, et al. Ocean, Cryosphere and Sea Level Change. In: Masson-Delmotte V, Zhai P, Pirani A, Connors SL, Péan C, Berger S, Caud N, et al., editors. Climate Change 2021: The Physical Science Basis. Contribution of Working Group I to the Sixth Assessment Report of the Intergovernmental Panel on Climate Change. Cambridge: Cambridge University Press. Forthcoming.

[104] Garner GG, Kopp RE, Hermans T, Slangen ABA, Koubbe G, Turilli M, et al. Framework for Assessing Changes To Sea-level (FACTS). Geoscientific Model Development. Forthcoming.

[105] Garner GG, Hermans T, Kopp RE, Slangen ABA, Edwards TL, Levermann A, et al. IPCC AR6 Sea-Level Rise Projections. Version 20210809. PO.DAAC, CA, USA. Dataset accessed 2022-02-23.

[106] Fan D, Nguyen DV, Su J, Bui VV, Tran DL. Coastal morphological changes in the Red River Delta under increasing natural and anthropic stresses. Anthropocene Coasts. 2019;2: 51–71. doi: 10.1139/anc-2018-0022

[107] Nguyen DV, Fan D, Bui VV, Tran DL. Sediment budget and morphological change in the Red River Delta under increasing human interferences. Mar Geol. 2021;431: 106379. doi: 10.1016/j.margeo.2020.106379

[108] Hoang PHY, Tran TTN, Tran N, Ngo QT, Hoang AK, Doan DL, et al. Late Pleistocene-Holocene sedimentary evolution in the coastal zone of the Red River Delta. Heliyon. 2021;7: e05872. doi: 10.1016/j.heliyon.2020.e05872 33521345 PMC7820486

[109] O’Donnell S, Nguyen TMH, Stimpson C, Holmes R, Kahlert T, Hill E. et al. Holocene development and human use of mangroves and limestone forest at an ancient *hong* lagoon in the Tràng An karst, Ninh Binh, Vietnam. Quaternary Sci Rev. 2020;242: 106416. doi: 10.1016/j.quascirev.2020.106416

[110] MONRE (Ministry of Natural Resources and Environment). Climate change and sea level rise scenarios for Viet Nam–Summary for policymakers; 2016.

[111] Ziegler AD, Lim HS, Wasson RJ, Williamson F. Flood mortality in SE Asia: Can palaeo-historical information help save lives? Hydrol Process. 2020;e13989: 1–8. doi: 10.1002/hyp.13989

[112] An Z, Porter SC, Kutzbach JE, Wu X, Wang S, Liu X, et al. Asynchronous Holocene optimum of the East Asian monsoon. Quat Sci Rev. 2000;19: 743–762.

[113] Yang X, Yang H, Wang B, Huang L-J, Shen C-C, Edwards RL, et al. Early-Holocene monsoon instability and climatic optimum recorded by Chinese stalagmites. The Holocene. 2019;29(6): 1059–1067. doi: 10.1177/09596836198314

[114] Lu F, Ma C, Zhu C, Lu H, Zhang X, Huang K, et al. Variability of East Asian summer monsoon precipitation during the Holocene and possible forcing mechanisms. Clim Dyn. 2018. doi: 10.1007/s00382-018-4175-6

[115] Wang SY, Lü HY, Liu JQ, Negendank JFW. The early Holocene optimum inferred from a high-resolution pollen record of Huguangyan Maar Lake in southern China. Chinese Sci Bull. 2007;52(20): 2829–2836. doi: 10.1007/s11434-007-0419-2

[116] Li Z, Pospelova V, Liu L, Zhou R, Song B. High-resolution palynological record of Holocene climatic and oceanographic changes in the northern South China Sea. Palaeogeogr Palaeoclimatol Palaeoecol. 2017;483: 94–124. doi: 10.1016/j.palaeo.2017.03.009

[117] Wang Y, Cheng H, Edwards RL, He Y, Kong X, An Z, et al. The Holocene Asian Monsoon: Links to Solar Changes and North Atlantic Climate. Science. 2005;308: 854–857. doi: 10.1126/science.1106296 15879216

[118] Wei G, Deng W, Yu K, Li X-h, Sun W, Zhao J-x. Sea surface temperature records in the northern South China Sea from mid-Holocene coral Sr/Ca ratios. Paleoceanography. 2007;22: PA3206. doi: 10.1029/2006PA001270

[119] Hu D, Böning P, Köhler CM, Hillier S, Pressling N, Wan S, et al. Deep sea records of the continental weathering and erosion response to East Asian monsoon intensification since 14 ka in the South China Sea. Chem Geol. 2012;326–237: 1–18. doi: 10.1016/j.chemgeo.2012.07.024

[120] Rabett R, Coward F, Van TT, Stimpson CM, Kahlert T, Bachtsevanidou Strantzali I, et al. Human Adaptation to Coastal Evolution: Late Quaternary evidence from Southeast Asia (SUNDASIA)–A report on the first year of the project. Tràng An Landscape Complex World Heritage Property, Ninh Binh Province, Vietnam. In: State of Conservation Report. 2017. Pp. 41–64. Available from: https://whc.unesco.org/document/165143

[121] Wu M-S, Zong Y, Mok K-M, Cheung K-M, Xiong H, Huang G. Holocene hydrological and sea surface temperature changes in the northern coast of the South China Sea. J Asian Earth Sci. 2017;135: 268–280. doi: 10.1016/j.jseaes.2017.01.004

[122] Zong Y, Huang K, Yu F, Zheng Z, Switzer A, Huang G, et al. The role of sea-level rise, monsoonal discharge and the palaeo-landscape in the early Holocene evolution of the Pearl River delta, southern China. Quat Sci Rev. 2012;54: 77–88. doi: 10.1016/j.quascirev.2012.01.002

[123] Bui DD, Kawamura A, Tong NT, Amaguchi H, Nakagawa N, Iseri Y. Identification of aquifer system in the whole Red River Delta, Vietnam. Geosci J. 2011;15(3): 323–338. doi: 10.1007/s12303-011-0024-x

[124] Tran TTT, Pham KH, Dao DB, Pham HA. Assessment the impact of climate change and sea level rise on the unconfined aquifer at the Red-River Delta of Vietnam: A case study at Thai Binh Province. In: Bui DT, Tran HT, Bui, X-N, editors. Proceedings of the International Conference on Innovations for Sustainable and Responsible Mining. ISRM 2020 –Volume 2. Cham: Springer; 2021. Pp. 326–348. Available from: https://link.springer.com/chapter/10.1007/978-3-030-60269-7_17

[125] Minh TN, Renaud FG, Sebesvari Z. Drivers of change and adaptation pathways of agricultural systems facing increased salinity intrusion in coastal areas of the Mekong and Red River deltas in Vietnam. Environ Sci Policy. 2019;92: 331–348. doi: 10.1016/j.envsci.2018.10.016

[126] Nguyen YTB, Kamoshita A, Dinh VTH, Matsuda H, Kurokura H. Salinity intrusion and rice production in Red River Delta under changing climate conditions. Paddy Water Environ. 2016. doi: 10.1007/s10333-016-0526-2

[127] Nguyen HN & Hop TBH. Vietnam–Vietnam in a time of El Niño. In: Glantz MH, editor. El Niño Ready Nations and Disaster Risk Reduction: 19 Countries in Perspective. Cham: Springer; 2022. Pp. 159–182. doi: 10.1007/978-3-030-86503-0

[128] Yuen KW, Tang TH, Vu DQ, Switzer AD, Teng P, Lee JSH. Interacting effects of land-use change and natural hazards on rice agriculture in the Mekong and Red River deltas in Vietnam. Nat. Hazards Earth Syst. Sci. 2021;21: 1473–1493. doi: 10.5194/nhess-21-1473-2021

[129] Grosjean G, Monteils F, Hamilton SD, Blaustein-Rejto D, Gatto M, Talsma T, et al. Increasing Resilience to Droughts in Vietnam; The Role of Forests, Agroforests and Climate Smart Agriculture. CCAFS- CIAT-UN-REDD Position Paper n. 1, Hanoi, Vietnam. 2016. Available from: https://cgspace.cgiar.org/

[130] Shao D, Mei Y, Yang Z, Wang Y, Yang W, Gao Y. Holocene ENSO variability in the South China Sea recorded by high-resolution oxygen isotope records from the shells of *Tridacna* spp. Sci Rep. 2020;10: 3921. doi: 10.1038/s41598-020-61013-2 32127633 PMC7054325

[131] Bui DD, Kawamura A, Tong NT, Amaguchi H, Nakagawa N. Spatio-temporal analysis of recent groundwater-level trends in the Red River Delta, Vietnam. Hydrogeol J. 2012;20: 1635–1650. doi: 10.1007/s10040-012-0889-4

[132] Tuan LA, Hoanh CT, Miller F, Sinh BT. Flood and salinity management in the Mekong Delta, Vietnam. In: Be TT, Sinh BT, Miller Feditors. Challenges to sustainable development in the Mekong Delta: Regional and national policy issues and research needs: Literature analysis. Bangkok, Thailand: The Sustainable Mekong Research Network (Sumernet); 2007. Pp.15–68.

[133] Nguyen VH, Tong NT, Nguyen DR, Trieu DH, Tong TT. Potential for the desalination of a brackish groundwater aquifer under a background of rising sea level via salt intrusion prevention river gates in the coastal area of the Red River Delta, Vietnam. Environ Dev Sustain. 2018;20: 2747–2771. doi: 10.1007/s10668-017-0014-x

[134] Tan L, Li Y, Wang X, Cai Y, Cheng H, Ma L, et al. Holocene monsoon change and abrupt events on the Western Chinese Loess Plateau as revealed by accurately dated stalagmites. Geophys Res Lett. 2020;46: e2020GL090273. doi: 10.1029/2020GL090273

[135] Sheng M, Wang X, Zhang S, Chu G, Su Y, Yang Z. A 20,000-year high-resolution pollen record from Huguangyan Maar Lake in tropical–subtropical South China. Palaeogeogr Palaeoclimatol Palaeoecol. 2017;472: 83–92. doi: 10.1016/j.palaeo.2017.01.038

[136] Tan L, Li Y, Han W. A Paleoclimate Prognosis of the Future Asian Summer Monsoon Variability. Atmosphere. 2021;12: 1391. doi: 10.3390/atmos12111391

[137] Beobide-Arsuaga G, Bayr, Reintges A, Latif M. Uncertainty of ENSO-amplitude projections in CMIP5 and CMIP6 models. Clim Dyn. 2021;56: 3875–3888. doi: 10.1007/s00382-021-05673-4

[138] Le T, Ha K-J, Bae D-H. Projected response of global runoff to El Niño-Southern oscillation. Environ Sci Lett. 2021;16: 084037. doi: 10.1088/1748-9326/ac13ed

[139] Krauss KW, McKee KL, Lovelock CE, Cahoon DR, Saintilan N, Reef R, et al. How mangrove forests adjust to rising sea level. New Phytol. 2014;202: 19–34. doi: 10.1111/nph.12605 24251960

[140] Saintilan N, Khan NS, Ashe E, Kelleway JJ, Rogers K, Woodroffe CD, et al. Thresholds of mangrove survival under rapid sea level rise. Science. 2020;368(6495): 1118–1121. doi: 10.1126/science.aba2656 32499441

[141] Nguyen TD, Nguyen THL, Nguyen TTC, Saito Y, Nguyen TMH, Nguyen TMP, et al. Holocene paleoshoreline changes of the Red River Delta, Vietnam. Rev Palaeobot Palyno. 2020;278: 104235. doi: 10.1016/j.revpalbo.2020.104235

[142] Funabiki A, Saito Y, Vu VP, Nguyen H, Haruyama S. Natural levees and human settlement in the Song Hong (Red River) delta, northern Vietnam. The Holocene. 2012;22(6): 637–648. doi: 10.1177/0959683611430847

[143] Li Z, Saito Y, Matsumotoa E, Wang Y, Tanabe S, Vu QL. Climate change and human impact on the Song Hong (Red River) Delta, Vietnam, during the Holocene. Quat Int. 2006;144: 4–28. doi: 10.1016/j.quaint.2005.05.008

[144] Tanabe S, Hori K, Saito Y, Haruyama S, Vu VP, Kitamura A. Song Hong (Red River) delta evolution related to millennium-scale Holocene sea-level changes. Quat Sci Rev. 2003;22(21): 2345–2361. doi: 10.1016/S0277-3791(03)00138-0

[145] Tanabe S, Saito Y, Vu QL, Hanebuth TJJ, Ngo QL, Kitamura A. Holocene evolution of the Song Hong (Red River) delta system, northern Vietnam. Sediment Geol. 2006;187: 29–61. doi: 10.1016/j.sedgeo.2005.12.004

[146] Yan S, Zhao J-x, Lau AYA, Roff G, Leonard ND, Clark TR, et al. Episodic Reef Growth in the Northern South China Sea linked to Warm Climate During the Past 7,000 Years: Potential for Future Coral Refugia. J Geophys Res Biogeosciences. 2020;124: 1032–1043. doi: 10.1029/ 2018JG004939

[147] Bui VV, Fan D, Nguyen DV, Tran DL, Tran DT, Hoang VL. Morphological Change in the Northern Red River Delta, Vietnam. J. Ocean Univ. China. 2018;17(6): 1272–1280. doi: 10.1007/s11802-018-3777-2

[148] Vinh VD, Ouillon S, Thanh TD, Chu LV. Impact of the Hoa Binh dam (Vietnam) on water and sediment budgets in the Red River basin and delta. Hydrol Earth Syst Sci. 2014;18: 3987–4005. doi: 10.5194/hess-18-3987-2014

[149] Doan DL & Boyd WE. Holocene Coastal Stratigraphy and the Sedimentary Development of the Hai Phong Area of the Bac Bo Plain (Red River Delta), Vietnam. Aust Geogr. 2003;34(2): 177–194. doi: 10.1080/00049180301737

[150] Tran VD, Tran DT, Nguyen VT. Monitoring coastal erosion in Red River Delta, Vietnam–A contribution from remote sensing data. Asian J Geoinfo. 2003;3(3): 73–78.

[151] Saito Y. Deltas in Southeast and East Asia: Their evolution and current problems. In: Mimura N & Yokoki H, editors. Global Change and Asia Pacific Coasts. Proceedings of APN/SURVAS/LOICZ Joint Conference on Coastal Impacts of Climate Change and Adaptation in the Asia-Pacific Region, APN, Kobe, Japan; 2000. Pp. 185–191.

[152] Watson J, Hamilton-Smith E, Gillieson D, Kiernan K. Guidelines for Cave and Karst Protection. IUCN World Commission on Protected Areas (WCPA). Gland and Cambridge: IUCN; 1997. 63pp.

[153] Li P, Li M, Gan H, Xi Z. A preliminary study on sediment records of possible typhoon in the northern South China Sea during the past 6500 yr. The Holocene. 2021;31(7): 1221–1228. doi: 10.1177/09596836211003

[154] Zhou L, Gao S, Jia J, Zhang Y, Yang Y, Mao L, et al. Extracting historic cyclone data from coastal dune deposits in eastern Hainan Island, China. Sediment Geol. 2019;392: 105524. doi: 10.1016/j.sedgeo.2019.105524

[155] Zhou L, Gao S, Jia J, Yang Y, Tong C, Wang A. Paleo-typhoon events as indicated by coral reef boulder deposits on the southern coast of Hainan Island, China. Front Mar Sci. 2021;8: 746773. doi: 10.3389/fmars.2021.746773

[156] Goff J, Witter R, Terry J, Spiske M. Palaeotsunamis in the Sino-Pacific region. Earth Sci Rev. 2020;210: 103352. doi: 10.1016/j.earscirev.2020.103352

[157] Rana A, Zhu Q, Detken A, Whalley K, Castet C. Strengthening climate-resilient development and transformation in Viet Nam. Clim Change. 2022;170: 23pp. doi: 10.1007/s10584-021-03290-y

[158] Giesen W, Wulffraat S, Zieren M, Scholten L. Mangrove Guidebook for Southeast Asia. FAO (Regional Office for Asia and the Pacific & Wetlands International, 2006). Bangkok: Dharmasarn Co., Ltd.; 2007. Available from: https://www.fao.org/3/ag132e/ag132e00.htm

[159] Turvey ST, Walsh C, Hansford JP, Crees JJ, Bielby J, Duncan C, et al. Complementarity, completeness and quality of long-term faunal archives in an Asian biodiversity hotspot. Philos T R Soc B. 2019;374: 20190217. doi: 10.1098/rstb.2019.0217 31679488 PMC6863502

[160] Lau WWY. Beyond carbon: Conceptualizing payments for ecosystem services in blue forests on carbon and other marine and coastal ecosystem services. Ocean Coast Manage. 2013;83: 5–14. doi: 10.1016/j.ocecoaman.2012.03.011

[161] Stewart HM & Swan S. Final evaluation of the UN-REDD Viet Nam Programme. Geneva: UN-REDD Programme; 2013. Available from: https://www.un-redd.org/document-library/un-redd-viet-nam-national-programme-final-evaluation-report-april-2013

[162] Pham TT, Bennett K, Phuong VT, Brunner J, Dung LN, Nguyen DT. Payments for forest environmental services in Vietnam: From policy to practice. CIFOR Occasional Paper 93. Bogor: Center for International Forestry Research; 2013.

[163] Talliardat P, Friess DA, Lupascu M. Mangrove blue carbon strategies for climate change mitigation are most effective at the national scale. Biol Letters. 2018;14: 20180251. doi: 10.1098/rsbl.2018.0251 30355678 PMC6227866

[164] Noon ML, Goldstein A, Ledezma JC, Roehrdanz PR, Cook-Patton SC, Spawn-Lee SA, et al. Mapping the irrecoverable carbon in Earth’s ecosystems. Nat Sustain. 2021. doi: 10.1038/s41893-021-00803-6

[165] MacNae W. A general account of the fauna and flora of mangrove swamps and forests in the Indo-West-Pacific region. Adv Mar Biol. 1968;6: 73–270. doi: 10.1016/S0065-2881(08)60438-1

[166] Orchard SE, Stringer LC, Quinn CH. Mangrove system dynamics in Southeast Asia: linking livelihoods and ecosystem services in Vietnam. Reg Environ Change. 2016;16: 865–879. doi: 10.1007/s10113-015-0802-5

[167] Hochard JP, Hamilton S, Barbier EB. Mangroves shelter coastal economic activity from cyclones. Proc Natl Acad Sci USA. 2019;116(25): 12232–12237. doi: 10.1073/pnas.1820067116 31160457 PMC6589649

[168] Nguyen HT, Adger WN, Kelly PM. Natural resource management in mitigating climate impacts: the example of mangrove restoration in Vietnam. Global Environ Chang. 1998;8(1): 49–61.

[169] Hong PN. Reforestation of mangrove after severe impacts of herbicides during the Viet Nam war: The case of Can Gio. Unasylva. 2001;207(52): 57–60. Available from: https://www.fao.org/3/y2795e/y2795e11.htm#o

[170] Veettil BK, Ward RD, Quang NX, Trang NTT, Giang TH. Mangroves of Vietnam: Historical development, current state of research and future threats. Estuar Coast Shelf S. 2019;218: 212–236. 10.1016/j.ecss.2018.12.021

[171] FAO (Food and Agriculture Organisation of the United Nations). The World’s Mangroves 1980–2005: A thematic study prepared in the framework of the Global Forest Resources Assessment 2005. FAO Forestry Paper 153; 2007.

[172] Hai NT, Dell B, Phuong VT, Harper RJ. Towards a more robust approach for the reforestation of mangroves in Vietnam. Ann For Sci. 2020;77: 18. doi: 10.1007/s13595-020-0921-0

[173] Powell N, Osbeck M, Sing BT, Vu CT. Mangrove restoration and rehabilitation for climate change adaptation in Vietnam. Washington DC: World Resources Report Case Study; 2011.

[174] Jhaveri NJ, Petrova S, Sommerville M, Le TVH, Nguyen NH. Red River Delta Coastal Spatial Planning and Mangrove Governance Assessment. Washington, DC: USAID Tenure and Global Climate Change Program; 2017.

[175] Pham TT & Nguyen VD. A decade of mangrove conservation achievements and challenges in Vietnam. Center for International Forestry Research (CIFOR) InfoBrief. 2021;337. doi: 10.17528/cifor/008099

[176] Blasco F, Gauquelin T, Rasolofoharinoro M, Denis J, Aizpuru M, Caldairou V. Recent advances in mangrove studies using remote sensing data. Mar Freshwater Res. 1998;49: 287–296. doi: 10.1071/MF97153

[177] Blasco F, Aizpuru M, Gers C. Depletion of the mangroves of Continental Asia. Wetl Ecol Manag. 2001;9: 245–256

[178] Brander LM, Wagtendonk AJ, Hussain SS, McVittie A, Verburg PH, de Groot R. et al. Ecosystem service values for mangrove in Southeast Asia: A meta-analysis and value transfer application. Ecosyst Serv. 2012;1: 62–69. doi: 10.1016/j.ecoser.2012.06.003

[179] Ministry of Agriculture and Rural Development (MARD) Coastal forest protection and development plan to response to climate change for the period 2015–2020 (approved at decision 120/QT-TTg dated 22nd January 2015 of Vietnamese Prime Minister). Hanoi: Ministry of Agriculture and Rural Development; 2014.

[180] Lewis RR III. Mangrove restoration–Costs and benefits of successful ecological restoration. In: Proceedings of the Mangrove Valuation Workshop, Universiti Sains Malaysia, Penang, 4–8 April 2001. Stockholm: Beijer International Institute of Ecological Economics; 2001. Available from: https://www.fao.org/forestry/10560-0fe87b898806287615fceb95a76f613cf.pdf

[181] IUCN Viet Nam national Strategy and Action Plan (2011–2013). Mangroves for the Future. Gland, Switzerland: IUCN; 2012. Available from: https://www.iucn.org/downloads/mff_vn_nsap_final_march_28_2011.pdf

[182] Nguyen NH. Cost-benefit analysis of climate adaptation: A case study of mangrove conservation and reforestation in Ca Mau Province, Vietnam. J Mekong Soc. 2015;11(2): 19–43.

[183] Maurand PL. Indochine forestière: les forets d’Indochine–exploitation–defrichement–amenagement–reconstitution des forets utilisation des bois–sous-produits forestiers. Hanoi: Impressions d’Extrême-Orient; 1943.

[184] Westing AH. The environmental aftermath of warfare in Viet Nam. Nat Resour J. 1983;23(2): 365–389. Available from: https://digitalrepository.unm.edu/cgi/viewcontent.cgi?article=2568&context=nrj

[185] Schmitt K & Duke NC. Mangrove management, assessment and monitoring. In: Pancel L & Köhl M, editors. Tropical Forestry Handbook. Berlin Heidelberg: Springer-Verlag; 2015. Pp. 1725–1759. doi: 10.1007/978-3-642-41554-8_126–1

[186] IUCN An appraisal of mangrove management in micro-tidal estuaries and lagoons in Sri Lanka. Gland, Switzerland: IUCN; 2011. Available from: https://portals.iucn.org/library/node/10197

[187] Joffre O & Schmitt K. A brief history of mangrove distribution and coastline development in Soc ‘Irang Province, Vietnam, to address coastal management strategies. In: Stewart MA & Coclanis PA, editors. Water and Power: Environmental Governance and Strategies for Sustainability in the Lower Mekong Basin. Advances in Global Change Research 64. Cham, Switzerland: Springer International Publishing AG; 2019. Pp. 67–85. doi: 10.1007/978–3–319–90400–9_5

[188] Birks HJB. Ecological palaeoecology and conservation biology: controversies, challenges, and compromises. Int J Biodivers Sci Ecosyst Serv Manag. 2012;8(4): 292–304. doi: 10.1080/21513732.2012.701667

[189] Gillson L & Marchant R. From myopia to clarity: sharpening the focus of ecosystem management through the lens of palaeoecology. Trends Ecol Evol. 2014;29(6): 317–325. doi: 10.1016/j.tree.2014.03.010 24768602

[190] Riedinger-Whitmore MA. Using palaeoecological and palaeoenvironmental records to guide restoration, conservation and adaptive management of Ramsar freshwater wetlands: lessons from the Everglades, USA. Mar Freshwater Res. 2016;67: 707–720. doi: 10.1071/MF14319

[191] Saunders KM & Gell PA. Paleoecological evidence for variability and change in estuaries: Insights for management. In: Weckström K, Saunders KM, Gell PA, Skilbeck CG, editors. Applications of Paleoenvironmental Techniques in Estuarine Studies., Developments in Paleoenvironmental Research 20. GX Dordrecht: Springer Nature; 2017. Pp. 75–87. doi: 10.1007/978-94-024-0990-1_4

[192] Weckström K, Saunders KM, Gell PA, Skilbeck CG (editors). Applications of Paleoenvironmental Techniques in Estuarine Studies., Developments in Paleoenvironmental Research 20. GX Dordrecht: Springer Nature; 2017. doi: 10.1007/978-94-024-0990-1_4

[193] Jhaveri N, Nguyen TD, Nguyen KD. Mangrove collaborative management in Vietnam and Asia. Global–Tenure and Global Climate Change, USAID; 2018. Available from: https://land-links.org/wp-content/uploads/2018/03/USAID_Land_Tenure_TGCC_Mangrove_Collaborative_Management_Vietnam_Asia.pdf

[194] Tran T, Nguyen VT, Nuynh TLH, Mai VK, Nguyen XH, Doan HP. Climate change and sea level rise scenarios for Viet Nam–Summary for policymakers. Ministry of Natural Resources and Environment; 2016.

[195] Rabett R, Coward F, Holmes R, Bachtsevanidou Strantzali I, Green E, Hill E. et al. Human adaptation to coastal evolution: late Quaternary evidence from Southeast Asia (SUNDASIA)–A report on the second year of the project. Viet Archaeol. 2019; 13: 23–48. Available from: https://sundasia.files.wordpress.com/2019/10/2nd-year-report_off-print_combined-front-matter-report-pages.pdf

[196] Marchand M. Mangrove restoration in Vietnam: key considerations and a practical guide. Deltares, Delft: Hanoi Water Resources University and Delft University of Technology; 2008. Available from: http://resolver.tudelft.nl/uuid:98b5ba43-1452-4631-81dc-ad043ef3992c

[197] Ellison AM. Mangrove restoration: Do we know enough? Restor Ecol. 2000;8(3): 219–229. Available from: https://harvardforest.fas.harvard.edu/sites/default/files/ellison-pubs/2000/ellison_2000.pdf

[198] Brown O, Crawford A, Hammill A. Natural disasters and resource rights building resilience, rebuilding lives. Manitoba: International Institute for Sustainable Development; 2006.

[199] Watkins C, Massey D, Brooks J, Ross K, Zellner ML. Understanding the mechanisms of collective decision making in ecological restoration: An Agent-Based Model of actors and organizations. Ecol Soc. 2013;18(2): 32. Available from: http://www.ecologyandsociety.org/vol18/iss2/art32/

[200] Wolf S, Pham M, Matthews N, Bubeck P. Understanding the implementation gap: policy-makers’ perceptions of ecosystem-based adaptation in Central Vietnam Clim Dev. 2020. doi: 10.1080/17565529.2020.1724068

[201] Gupta H, Kao S.-J, Dai M. The role of mega dams in reducing sediment fluxes: A case study of large Asian rivers. J Hydrol. 2012;464–465: 447–458. doi: 10.1016/j.jhydrol.2012.07.038

[202] Mason C, Hobday AJ, Alderman R, Lea M-A. Climate adaptation interventions for iconic fauna. Conserv Sci Prac. 2021;3(7): e434. doi: 10.1111/csp2.434

[203] Rogers HS, Donoso I, Traveset A, Fricke EC. Cascading impacts of seed disperser loss on plant communities and ecosystems. Annu Rev Ecol Evol Syst. 2021;52: 641–66. doi: 10.1146/annurev-ecolsys-012221-111742

[204] Berzaghi F, Verbeeck H, Nielsen MR, Doughty CE, Bretagnolle F, Marchetti M, et al. Assessing the role of megafauna in tropical forest ecosystems and biogeochemical cycles–the potential of vegetation models. Ecography. 2018;41: 1934–1054. doi: 10.1111/ecog.03309

[205] Howe H F & Smallwood J. Ecology of seed dispersal. Annu Rev Ecol Syst. 1982;13: 201–228.

[206] Putz FE. Aseasonality in Malaysian tree Phenology. Malays. 1979;42(1): 1–24.

[207] Fricke EC, Ordonez A, Rogers HS, Svenning J-C. The effects of defaunation on plants’ capacity to track climate change. Science. 2022;375: 210–214. doi: 10.1126/science.abk3510 35025640

[208] IUCN Global standard for nature-based solutions. A user-friendly framework for the verification, design and scaling up of NbS. Gland, Switzerland: IUCN; 2020. doi: 10.2305/IUCN.CH.2020.08.en

[209] Williams PJ. Tropical plant-animal interactions: Linking defaunation with seed predation, and resource- dependent co-occurrence. PhD thesis, University of Montana, Graduate Student Theses, Dissertations, & Professional Papers. 11777. 2021. Available from: https://scholarworks.umt.edu/etd/11777

[210] Hale SL & Koprowski JL. Ecosystem-level effects of keystone species reintroduction: a literature review. Restor Ecol. 2018. doi: 10.1111/rec.12684

[211] Krause T & Tilker A. How the loss of forest fauna undermines the achievement of the SDGs. Ambio. 2022;51: 103–113. doi: 10.1007/s13280-021-01547-5 33825158 PMC8023557

[212] Williams PJ, Ong RC, Brodie JF, Luskin MS. Fungi and insects compensate for lost vertebrate seed predation in an experimentally defaunated tropical forest. Nat Comm. 2021;12: 1650. doi: 10.1038/s41467-021-21978-8 33712621 PMC7955059

[213] Muller-Landau HC. Predicting the long-term effects of hunting on plant species composition and diversity in tropical forests. Biotropica. 2007;39(3): 372–384. doi: 10.1111/j.1744-7429.2007.00290.x

[214] Clements R, Sodhi NS, Schilthuizen M, Ng PKL. Limestone karsts of Southeast Asia: imperiled arks of biodiversity. Bioscience. 2006;56(9): 733–742. doi: 10.1641/0006-3568(2006)56733:LKOSAI2.0.CO;2

[215] Duckworth JW, Batters G, Belant JL, Bennett EL, Brunner J, Burton J. et al. Why South-east Asia should be the world’s priority for averting imminent species extinctions, and a call to join a developing cross-institutional programme to tackle this urgent issue. SAPIENS: Surveys and Perspectives Integrating Environment and Society. 2012;5(2): 77–95. Available from: https://www.researchgate.net/publication/235248971

[216] Furey NM, Mackie IJ, Racey PA. Bat diversity in Vietnamese limestone karst areas and the implications of forest degradation. Biodiv Conserv. 2010;19: 1821–1838. doi: 10.1007/s10531-010-9806-0

[217] Milner-Gulland EJ, Bennett EL, and the SCB 2002 Annual Meeting Wild Meat Group. Wild meat: the bigger picture. Trends Ecol Evol. 2003;18(7): 351–357. doi: 10.1016/S0169-5347(03)00123-X

[218] Sodhi NS, Koh PL, Brook BW, Ng, PKL. Southeast Asian biodiversity: an impending disaster. Trends Ecol Evol. 2004;19(12): 654–660. doi: 10.1016/j.tree.2004.09.006 16701328

[219] Sodhi NS, Koh LP, Clements R, Wanger TC, Hill JK, Hamer KC. Et al. Conserving Southeast Asian forest biodiversity in human-modified landscapes. Biol Conserv. 2010;143: 2375–2384. doi: 10.1016/j.biocon.2009.12.029

[220] Vermeulen J & Whitten T. Biodiversity and Cultural Property in the Management of Limestone Resources: Lessons from East Asia. The World Bank; 1999.

[221] Gao Y, Bin Ai, Kong H, Kang M, Huang H. Geographical pattern of isolation and diversification in karst habitat islands: a case study in the *Primulina burnean* complex. J Biogeogr. 2015;42: 2131–44. doi: 10.1111/jbi.12576

[222] Hughes A. Understanding the drivers of Southeast Asian biodiversity loss. Ecosphere. 2017;8(1): e01624. doi: 10.1002/ecs2.1624

[223] Tuyet D. Characteristics of karst ecosystems of Vietnam and their vulnerability to human impact. Acta Geol Sin-engl. 2001;75(3): 325–29. doi: 10.1111/j.1755-6724.2001.tb00539.x

[224] Tuyet D, Tran VT, Pham KT. Characteristics of humid tropical karst of Vietnam. In: Batelaan O, Dusar M, Masschelein J, Vu TT, Tran VT, Nguyen XK, editors. Trans-KARST 2004: Proceedings of the International Transdisciplinary Conference on Development and Conservation of Karst Regions. Hanoi: Institute of Geology and Mineral Resources; 2004. Pp. 240–249. Available from: http://st1.asflib.net/MEDIA/ASF-CD/ASF-M-00008/Trans-KARST2004Proceedings.pdf#page=250

[225] Tran CT. From Ha Long Bay to Trang An Landscape Complex: Issues on tourism management at World Heritage Sites, Vietnam. SPAFA journal. 2019;3. Pp. 13. doi: 10.26721/spafajournal.v3i0.607

[226] Hoang T & Pulliat G. Green for whom? Exploring ecotourism as a climate-adaptation strategy in Tràng An, Vietnam. In: Daniere AG & Garschagen M, editors. Urban Climate Resilience in Southeast Asia. Cham: Springer Nature Switzerland; 2019. Pp. 179–199. doi: 10.1007/978-3-319-98968-6_9

[227] Stimpson C, O’Donnell S, Nguyen TMH, Holmes R, Utting B, Kahlert T. et al. Confirmed archaeological evidence of water deer in Vietnam: relics of the Pleistocene or a shifting baseline? Roy Soc Open Sci. 2021;8: 210529. doi: 10.1098/rsos.210529 34234958 PMC8242832

[228] IUCN Guidelines for Re-Introductions. Prepared by the IUCN/SSC Re-Introduction Specialist Group. Gland, Switzerland and Cambridge UK: IUCN; 1998. Available from: https://www.iucn.org/content/iucn-guidelines-re-introductions

[229] Rabett R, Coward F, Holmes R, Tran VT, Nguyen TD, Bui VM, et al. Human adaptation to coastal evolution: late Quaternary evidence from Southeast Asia (SUNDASIA)–A report on the third year of the project. Viet Archaeol. 2021;15: 33–63 (2021). Available from: https://sundasia.files.wordpress.com/2021/12/sundasia_3rd-year-project-report_off-print.pdf

[230] Corlett RT. Frugivory and seed dispersal by vertebrates in the Oriental (Indomalayan) Region. Biol Rev. 1998;73: 413–448. doi: 10.1017/s0006323198005234 9951414

[231] Corlett RT. Frugivory and seed dispersal by vertebrates in tropical and subtropical Asia: An update. Glob Ecol Conserv. 2017;11: 1–22. doi: 10.1016/j.gecco.2017.04.007

[232] Pérez-Méndez N, Jordano P, García C, Valido A. The signatures of Anthropocene defaunation: cascading effects of the seed dispersal collapse. Sci Rep. 2016;6: 24820. doi: 10.1038/srep24820 27091677 PMC4835773

[233] Corlett RT. The impact of hunting on the mammalian fauna of tropical Asian forests. Biotropica. 2007;39(3): 292–303. doi: 10.1111/j.1744-7429.2007.00271.x

[234] Zhou Y, Chen W, Buesching CD, Newman C, Kaneko Y, Xiang M, et al. Hog badger (*Arctonyx collaris*) latrine use in relation to food abundance: evidence of the scarce factor paradox. Ecosphere. 2015a;6(1): 19. doi: 10.1890/ES14-00155.1(2015a)

[235] Zhou Y, Chen W, Kaneko Y, Newman C, Lioa Z, Zhu X, et al. Seasonal dietary shifts and food resource exploitation by the hog badger (*Arctonyx collaris*) in a Chinese subtropical forest. Eur J Wildl Res. 2015b;61: 125–133. doi: 10.1007/s10344-014-0881-5

[236] Duckworth JW & Nettelbeck AR. Observations of SmaIl-toothed Palm Civets Arctogalidia trivirgata in Khao Yai National Park: Notes on Feeding Techniques. Nat Hist Bul Siam Soc.2007;55(1): 187–192.

[237] Nakabayashi M, Inoue Y, Ahmad AH, Izawa M. Limited directed seed dispersal in the canopy as one of the determinants of the low hemi-epiphytic figs’ recruitments in Bornean rainforests. PLoS ONE.2019;14(6): e0217590. doi: 10.1371/journal.pone.0217590 31194749 PMC6564369

[238] Davis NE, Forsyth DM, Coulson G. Facilitative interactions between an exotic mammal and native and exotic plants: hog deer (*Axis porcinus*) as seed dispersers in south-eastern Australia. Biol Invasions. 2010;12: 1079–1092. doi: 10.1007/s10530-009-9525-1(2010)

[239] Lee S-K & Lee EJ. Internationally vulnerable Korean water deer (*Hydropotes inermis argyropus*) can act as an ecological filter by endozoochory. Global Ecol Conserv. 2020;24: e01368. doi: 10.1016/j.gecco.2020.e01368

[240] Bátori Z, Vojtkó A, Maák I-e, Lőrinczi G, Farkas T, Kántor N, et al. Karst dolines provide diverse microhabitats for different functional groups in multiple phyla. Sci Rep. 2019;9: 7176. doi: 10.1038/s41598-019-43603-x 31073136 PMC6509348

[241] Harrison S & Noss R. Endemism hotspots are linked to stable climatic refugia. Ann Bot. 2017;119(2): 207–214. doi: 10.1093/aob/mcw248 28064195 PMC5321063

[242] Trew BT & Maclean IMD. Vulnerability of global biodiversity hotspots to climate change. Global Ecol Biogeogr. 2021;30: 768–783. doi: 10.1111/geb.13272

[243] Rabett R, Ludgate N, Stimpson C, Hill E, Hunt C, Ceron J. et al. Tropical limestone forest resilience and late Pleistocene foraging during MIS-2 in the Tràng An massif, Vietnam. Quatern Int. 2017;448: 62–81. doi: 10.1016/j.quaint.2016.06.010

[244] Genes L & Dirzo R. Restoration of plant-animal interactions in terrestrial ecosystems. Biol Conserv. 2022;265: 109393. doi: 10.1016/j.biocon.2021.109393

[245] Stimpson CM, Utting B, O’Donnell S, Nguyen TMH, Kahlert T, Bui VM. et al. An 11 000-year-old giant muntjac subfossil from Northern Vietnam: implications for past and present populations. Roy Soc Open Sci. 2019;6(3): 181461. doi: 10.1098/rsos.181461 31032005 PMC6458398

[246] Ceron, J. L. R. A study of Late Pleistocene and Early Holocene macro-botanical remains from Hang Boi and Hang Trống cave sites in northern Việt Nam: Towards an understanding of landscape and human-plant utilisation. Master’s dissertation, Archaeological Studies Program, University of the Philippines. 2013. Available from: https://www.researchgate.net/publication/316165069

[247] IUCN/SSC Guidelines for Reintroductions and Other Conservation Translocations. Version 1.0. Gland, Switzerland and Cambridge UK: IUCN Species Survival Commission; 2013. Available from: https://www.iucn.org/content/guidelines-reintroductions-and-other-conservation-translocations

[248] Crees JJ & Turvey ST. What constitutes a ‘native’ species? Insights from the Quaternary faunal record. Biol Conserv. 2015;186: 143–148. doi: 10.1016/j.biocon.2015.03.007

[249] Louys J, Corlett RT, Price GJ, Hawkins S, Piper PJ. Rewilding the tropics, and other conservation translocations strategies in the tropical Asia-Pacific region. Ecol Evol. 2014;4(22): 4380–4398. doi: 10.1002/ece3.1287 25540698 PMC4267875

[250] Seddon PJ, Griffiths CJ, Soorae PS, Armstrong DP. Reversing defaunation: Restoring species in a changing world. Science. 2014;345(6195): 406–412. doi: 10.1126/science.1251818 25061203

[251] Galetti M, Pires AS, Brancalion PHS, Fernandez FAS. Reversing defaunation by trophic rewilding in empty forests. Biotropica. 2016;49(1): 5–8. doi: 10.1111/btp.12407

[252] Svenning J-G, Pedersen PBM, Donlan CJ, Ejmæs R, Faurby S, Galetti M, et al. Science for a wilder Anthropocene: Synthesis and future directions for trophic rewilding research. Proc Natl Acad Sci USA. 2016;113(4): 898–906. doi: 10.1073/pnas.1502556112 26504218 PMC4743824

[253] Hall CM. Tourism and rewilding: An introduction–definition, issues and review. J Ecotourism. 2019;18(4): 297–308. doi: 10.1080/14724049.2019.1689988

[254] Hayward MW, Scanlon RJ, Callen A, Howell LG, Klop-Toker KL, Di Blanco Y. et al. Reintroducing rewilding to restoration–Rejecting the search for novelty. Biol Conserv. 2019;233: 255–259. doi: 10.1016/j.biocon.2019.03.011

[255] Pettorelli N, Barlow J, Stephens PA, Durant SM, Connor B, Schulte to Bühne H. et al. Making rewilding fit for policy. J Appl Ecol. 2017;55: 1114–1125. doi: 10.1111/1365-2664.13082

[256] Carver S, Convery I, Hawkins S, Beyers R, Eagle A, Kun Z. et al. Guiding principles for rewilding. Conserv Biol. 2021;35(6): 1882–1893. doi: 10.1111/cobi.13730 33728690

[257] Piper PJ & Cranbrook EO. The potential for large protected areas for the secure re-introduction of Borneo’s lost ‘Megafauna’: A case for the Malay tapir *Tapirus indicus*. In: Stuebing RB, Unggang J, Ferner J, Ferner J, Giman K, Ping KK, editors. Proceedings of the regional conference: biodiversity conservation in tropical planted forests in South East Asia. Kuching: Forest Department, Sarawak Forest Corporation & Grand Perfect Sdn Bhd. 2007. Pp. 161–168.

[258] Steinmetz R, Chutipong W, Seuaturien N, Chirngsaard E, Khaengkhetkarn M. Population recovery patterns of Southeast Asian ungulates after poaching. Biol Conserv. 2010;143: 42–51. doi: 10.1016/j.biocon.2009.08.023

[259] Timmins RJ, Duckworth JW, Robichaud W, Long B, Gray TNE, Tilker A. *Muntiacus vuquangensis*. The IUCN Red List of Threatened Species. 2016: e.T44703A22153828. doi: 10.2305/IUCN.UK.2016-2.RLTS.T44703A22153828.en

[260] McCullough DR, Pei KCJ, Wang Y. Home range, activity patterns, and habitat relations of Reeves’ muntjacs in Taiwan. J Wildlife Manage. 2000;64(2): 430–441.

[261] Odden M & Wegge P. Predicting spacing behavior and mating systems of solitary cervids: A study of hog deer and Indian muntjac. Zoology. 2007;110: 261–270. doi: 10.1016/j.zool.2007.03.003 17614268

[262] Tilker A, Abrams JF, An N, Hörig L, Axtner J, Louvrier J, et al. Identifying conservation priorities in a defaunated tropical biodiversity hotspot. *Divers Distrib*. 2020;26: 426–440. doi: 10.1111/ddi.13029

[263] Nguyen AT, Tilker A, Nguyen T, Le M. Camera-trap records of muntjac in the lowlands of Hue Saola Nature Reserve, central Vietnam. Deer Specialist Group Newsletter. 2021;32: 37–47. Available from: http://www.icneotropical.org/archivos%20DEER/news/DSGNews32.pdf

[264] Stone R. Dam project reveals secret sanctuary of vanishing deer. Science. 2009;325: 1192. doi: 10.1126/science.325_1192b 19729627

[265] World Land Trust. Conservation win as new nature reserve in Vietnam gives another chance to extinction-facing ‘Asian Unicorn’. Press release 29.09.2020. Available from: https://www.worldlandtrust.org/wp-content/uploads/2020/09/SEPT20-PR-Khe-Nuoc-Trong.pdf

[266] Tilker A. Formation of a large-antlered muntjac working group and muntjac partnership. Deer Specialist Group Newsletter. 2018;30: 22–23. Available from: http://www.icneotropical.org/archivos%20DEER/news/DSGNews30.pdf

[267] Turvey ST, Hansford J, Brace S, Mullin V, Gu S, Sun G. Holocene range collapse of giant muntjacs and pseudo-endemism in the Annamite large mammal fauna. J Biogeogr. 2016;43: 2250–2260. doi: 10.1111/jbi.12763

[268] Chen M, Liu C, He X, Pei E, Yuan X, Zhang E. The efforts to re-establish the Chinese water deer population in Shanghai, China. Anim Prod Sci. 2016;56: 941–945. doi: 10.1071/AN14910

[269] Kim BJ, Oh DH, Chun SH, Lee SD. Distribution, density, and habitat use of the Korean water deer (*Hydropotes inermis argyropus*) in Korea. Landsc Ecol Eng. 2011;7(2): 291–297. doi: 10.1007/s11355-010-0127-y

[270] Harris RB & Duckworth JW. *Hydropotes inermis*. The IUCN Red List of Threatened Species. 2015: e.T10329A22163569. doi: 10.2305/IUCN.UK.2015- 2.RLTS.T10329A22163569.en

[271] Chen M, Pu A, He X, Zhang E, Ding Y, Wang T. et al. Chinese Water Deer (*Hydropotes inermis*) Reintroduction in Nanhui, Shanghai, China. Pakistan J Zool. 2015;47(5): 1499–1501.

[272] He X, Chen M, Zhang E. Home range of reintroduced Chinese water deer in Nanhui East Shoal Wildlife Sanctuary of Shanghai, China. Anim Prod. Sci. 2016;56: 988–996. doi: 10.1071/AN14858

[273] Schilling A-M, Rössner GE. The (sleeping) Beauty in the Beast–a review on the water deer, *Hydropotes inermis*. Hystrix. 2017;28(2): 121–133. doi: 10.4404/hystrix-28.2–12362

[274] Corlett RT. The shifted baseline: Prehistoric defaunation in the tropics and its consequences for biodiversity conservation. Biol. Conserv. 2013;163: 13–21. doi: 10.1016/j.biocon.2012.11.012

[275] Beguin J, Tremblay J-P, Thiffault N, Pothier D, Côté SD. Management of forest regeneration in boreal and temperate deer–forest systems: challenges, guidelines, and research gaps. Ecosphere. 2016;7(10): e01488. doi: 10.1002/ecs2.1488

[276] Zamboni T, Di Martino S, Jiménez-Pérez I. A review of a multispecies reintroduction to restore a large ecosystem: The Iberá Rewilding Program (Argentina). Perspect Ecol Conser. 2017;15: 248–256. doi: 10.1016/j.pecon.2017.10.001

[277] Nuttall MN, Griffin O, Fewster RM, McGoven PJK, Abernethy K, O’Kelly H, et al. Long-term monitoring of wildlife populations for protected area management in Southeast Asia. Conserv Sci Pract. 2021. E614. doi: 10.1111/csp2.614

[278] Andrade GSM & Rhodes JR. Protected areas and local communities: an inevitable partnership toward successful conservation strategies? Ecol Soc. 2012;17(4): 14. doi: 10.5751/ES-05216-170414

[279] Hofmann RR. Evolutionary steps of ecophysiological adaptation and diversification of ruminants: a comparative view of their digestive system. Oecologia. 1989;78: 443–457. doi: 10.1007/BF00378733 28312172

[280] Sridhara S, McConkey K, Prasad S, Corlett RT. Frugivory and seed dispersal by large herbivores of Asia. In: Ahrestani FS & Sankaran M, editors. The Ecology of Large Herbivores in South and Southeast Asia, Ecological Studies (Analysis and Synthesis), vol 225. Dordrecht: Springer Science+Business Media; 2016. Pp. 121–150. doi: 10.1007/978-94-017-7570-0_5

[281] Thinley P, Lassoie J, Morreale SJ, Curtis PD, Rajaratnam R, Vernes K, et al. High relative abundance of wild ungulates near agricultural croplands in a livestock-dominated landscape in Western Bhutan: Implications for crop damage and protection. Agr Ecosyst Environ. 2017;248: 88–95. doi: 10.1016/j.agee.2017.07.036

[282] Brodie JF, Helmy OE, Brockelman WY, Maron JL. Bushmeat poaching reduces the seed dispersal and population growth rate of a mammal-dispersed tree. Ecol Appl. 2009;19(4): 854–863. doi: 10.1890/08-0955.1 19544729

[283] Chen Deng, Bai Yang, Chen Liu, et al. Fruit characteristics and *Muntiacus muntijak vaginalis* (Muntjac) visits to individual plants of Choerospondias axillaris. Biotropica. 2001;33(4): 718–722.

[284] Ilyas O & Khan JA. Food habits of barking deer (*Muntiacus muntjak*) and goral (*Naemorhedus goral*) in Binsar Wildlife Sanctuary, India. Mammalia. 2003;67(4): 521–531. doi: 10.1515/mamm-2003-0406

[285] Kumar NS, Karanth KU, Nichols JD, Vaidyanathan S, Gardner B, Krishnaswamy J. Spatial dynamics and ecology of large ungulate populations in tropical forests of India. Singapore: Springer Nature Pte Ltd.; 2021. doi: 10.1007/978-981-15-6934-0

[286] Kim BJ & Lee S-D. Home range study of the Korean water deer (*Hydropotes inermis agyropus*) using radio and GPS tracking in South Korea: comparison of daily and seasonal habitat use pattern. J. Ecol. Field Biol. 2011;34(4): 365–370. doi: 10.5141/JEFB.2011.038

[287] Lee S-K, Shin W-J, Ahn S, Kim Y, Kim J-T, Lee EJ. Seed recovery and germination rate after gut passage by Korean water deer (*Hydropotes inermis argyropus*). Seed Sci Res. 2021;31: 311–318. doi: 10.1017/S0960258521000246

[288] Nadler T. The critical status of the Delacour’s langur (*Trachypithecus delacouri*) and the call for a National Action Plan. Viet J Primotol. 2015;2(4): 1–12 (2015). Available from: http://www.primate-sg.org/storage/pdf/VJP_2-4_pp1-12.pdf

[289] Workman C. Diet of the Delacour’s langur (*Trachypithecus delacouri*) in Van Long Nature Reserve, Vietnam. Am J Primatol. 2010;72(4): 317–324. doi: 10.1002/ajp.20785 20027639

[290] Workman C & Schmitt D. Positional behavior of Delacour’s Langurs (*Trachypithecus delacouri*) in northern Vietnam. Int J Primatol. 2012;33: 19–37. doi: 10.1007/s10764-011-9547-2

[291] Nguyen VL, Mai VQ, Nadler T. Rapid population increase of the ‘Critically Endangered’ Delacour’s langur (*Trachypithecus delacouri*) in Van Long Nature Reserve due to strict protection. Viet J Primatol. 2019;3(1): 3–18. Available from: http://www.primate-sg.org/storage/pdf/VJP_3_1_pp3-18.pdf

[292] Nadler T, Rabett R, O’Donnell S, Nguyen TMH. Delacour’s langur (*Trachypithecus delacouri*) reintroduction program: A preliminary report on the trial release into the Trang An UNESCO World Heritage Site, Ninh Binh Province, Vietnam. Viet J Primatol. 2020;3(2): 39–48. Available from: http://www.primate-sg.org/storage/pdf/VJP_3_2_pp39-48.pdf

[293] Nadler T, Quyet LK, Rawson BM, Coudrat CNZ. *Trachypithecus delacouri*. The IUCN Red List of Threatened Species 2020: e.T22043A17958988. doi: 10.2305/IUCN.UK.2020-2.RLTS.T22043A17958988.en

[294] The Prime Minister of Government Decision No: 628/QĐ-TTg: Approving urgent conservation action plan for primates in Vietnam to 2025, vision to 2030 (2017). Viet J Primatol. 2021;3(3): 9–94. Available from: http://www.primate-sg.org/storage/pdf/VJP_3_pp9-93.pdf

[295] Nguyen D. Van Long Nature Reserve Vietnam. Report commissioned by the Centre for Sustainable Development. IUCN. 2008. Available from: https://www.iccaconsortium.org/wp-content/uploads/2015/08/grassroot-vietnam-val-long-2008.pdf

[296] Blair ME, Nguyen TA, Le MD, Liu Z, Meng T, Horning N, et al. Karst as an abiotic driver of François’ langur distribution, with predictions for biological communities on karst under climate change. Front Biogeogr. 2022:14.1: e51838. doi: 10.21425/F5FBG51838

[297] Liu Z, Zhang L, Yan Z, Ren Z, Han F, Tan X, et al. Genomic mechanisms of physiological and morphological adaptations of limestone langurs to karst habitats. Mol Biol Evol. 2019;37(4): 952‐968. doi: 10.1093/molbev/msz301 31846031

[298] Chapman CA, Bonnell TR, Gogarten JF, Lambert JE, Omeja PA, Twinomugisha D, et al. Are primates ecosystem engineers? Int J Primatol. 2013;34: 1–14. doi: 10.1007/s10764-012-9645-9

[299] Haywood AM, Valdes PJ, Aze A, Barlow N, Burke A, Dolan AM, et al. What can palaeoclimate modelling do for you? Earth Syst Environ. 2019;3: 1–18. doi: 10.1007/s41748-019-00093-1

[300] Refsgaard JC, Madsen H, Andréassian V, Arnbjerg-Nielsen K, Davidson TA, Dreas M, et al. A framework for testing the ability of models to project climate change and its impacts. Climatic Change. 2014;122: 271–282. doi: 10.1007/s10584-013-0990-2

[301] Ta TKO, Nguyen VL, Saito Y, Gugliotta M, Tamura T, Nguyen TML, et al. Latest Pleistocene to Holocene stratigraphic record and evolution of the Paleo-Mekong incised valley, Vietnam. Mar Geol. 2021;106406. doi: 10.1016/j.margeo.2020.106406

[302] Phach PV, Laib VC, Shakirovc RB, Le DA, Tung DX. Tectonic Activities and Evolution of the Red River Delta (North Viet Nam) in the Holocene. Geotectonics. 2020;54(1): 113–129. doi: 10.1134/S0016852120010094

[303] Khan NS, Horton BP, Engelhart S, Rovere A, Vacchi M, Ashe EL, et al. Inception of a global atlas of sea levels since the Last Glacial Maximum. Quat Sci Rev. 2019;220: 359–71. doi: 10.1016/j.quascirev.2019.07.016

[304] Fan D, Guo Y, Wang P, Shi JZ. Cross-shore variations in morphodynamic processes of an open-coast mudflat in the Changjiang Delta, China: With an emphasis on storm impacts. Cont Shelf Res. 2006;26: 517–538. doi: 10.1016/j.csr.2005.12.011

[305] IUCN Ecosystem-based Adaptation: A natural response to climate change. Gland, Switzerland: IUCN; 2009. Available from: https://www.iucn.org/content/ecosystem-based-adaptation-a-natural-response-climate-change

[306] IUCN Cost and benefits of ecosystem based adaptation: The case of the Philippines. Gland, Switzerland: IUCN; 2016. Available from: https://portals.iucn.org/library/sites/library/files/documents/2016-009.pdf

[307] Secretariat of the Convention on Biological Diversity. Connecting biodiversity and climate change mitigation and Adaptation: Report of the second Ad Hoc technical expert group on biodiversity and climate change. Montreal: Secretariat of the Convention on Biological Diversity. 2009. https://www.cbd.int/doc/publications/ahteg-brochure-en.pdf

[308] Donatti CI, Harvey CA, Hole D, Panfil SN, Schurman H. Indicators to measure the climate change adaptation outcomes of ecosystem-based adaptation. Clim Change. 2020;158: 413–433. doi: 10.1007/s10584-019-02565-9

[309] Amano N, Wang WV, Boivin N, Roberts P. ‘Emptying Forests?’ Conservation Implications of Past Human–Primate Interactions. Trends Ecol Evol. 2021;36(4): 345–359. doi: 10.1016/j.tree.2020.12.004 33431163

[310] Redford KH. The empty forest. Bioscience. 1992;42(6): 412–422.

[311] Wilkie DS, Bennett EL, Peres CA, Cunningham AA. The empty forest revisited. Ann NY Acad. 2011;1223: 120–128. doi: 10.1111/j.1749-6632.2010.05908.x 21449969

[312] Dirzo R, Young HS, Galetti M, Ceballos G, Isaac NJB, Collen B. Defaunation in the Anthropocene. Science. 2014;345(6195): 401–406. doi: 10.1126/science.1251817 25061202

[313] Thorpe AS & Stanley AG. Determining appropriate goals for restoration of imperilled communities and species. J Appl Ecol. 2011;48: 275–79. doi: 10.1111/j.1365-2664.2011.01972.x

[314] Strang K & Rusi N. The challenges of conserving biodiversity: A spotlight on Southeast Asia. In: Underkoffler SC & Adams HR, editors. Wildlife Biodiversity Conservation: Multidisciplinary and Forensic Approaches. Cham: Springer; 2021. pp. 47–66. doi: 10.1007/978-3-030-64682-0

[315] Tang CQ, Matsui T, Ohashi H, Dong Y-F, Momohara A, Herrando-Moraira S, et al. Identifying long-term stable refugia for relict plant species in East Asia. Nature Comm. 2018;9: 4488. doi: 10.1038/s41467-018-06837-3 30367062 PMC6203703

[316] Hunt CO & Rabett R. Holocene landscape intervention and plant food production strategies in Island and Mainland SE Asia. J Archaeol Sci. 2014;51: 22–33. doi: 10.1016/j.jas.2013.12.011

[317] Levis C, Costa FRC, Bongers F, Peña-Claros M, Clement CR, Junqueira AB. et al. Persistent effects of pre-Columbian plant domestication on Amazonian forest composition. Science. 2017;355(6328): 925–931. doi: 10.1126/science.aal0157 28254935

[318] Lombardo U, Iriarte J, Hilbert L, Ruiz-Pérez J, Capriles JM, Veit H. Early Holocene crop cultivation and landscape modification in Amazonia. Nature. 2020;581: 190–193. doi: 10.1038/s41586-020-2162-7 32404996 PMC7250647

[319] Andermann T, Faurby S, Turvey ST, Antonelli A, Silvestro D. The past and future human impact on mammalian diversity. Sci Adv. 2020;6(36): eabb2313. doi: 10.1126/sciadv.abb2313 32917612 PMC7473673

[320] Jones RK, Piper PJ, Groves CP, Tuan NA, Mai Huong NT, Hao NT. et al. Shifting subsistence patterns from the Terminal Pleistocene to Late Holocene: A regional Southeast Asian analysis. Quatern Int. 2019;529: 47–56. doi: 10.1016/j.quaint.2019.01.006

[321] Roberts P, Hamilton R, Piperno DR. Tropical forests as key sites of the “Anthropocene”: Past and present perspectives. Proc Natl Acad Sci USA. 2021;118(40): e2109243118. doi: 10.1073/pnas.2109243118 34580229 PMC8501787

[322] Steffen W, Crutzen PJ, McNeill JR. The Anthropocene: Are humans now overwhelming the great forces of Nature? Ambio. 2007;36(8): 614–621. doi: 10.1579/0044-7447(2007)36[614:taahno]2.0.co;2 18240674

[323] Wyborn C, Datta A, Montana J, Ryan M, Leith P, Caffin B, et al. Co-producing sustainability: Reordering the governance of science, policy, and practice. Ann Rev Env Resour. 2019;44: 319–346. doi: 10.1146/annurev-environ-101718-033103

[324] Young JC, Waylen KA, Sarkki S, Albon S, Bainbridge I, Balian E, et al. Improving the science-policy dialogue to meet the challenges of biodiversity conservation: having conversations rather than talking at one-another. Biodivers Conserv. 2014; 23: 387–404. doi: 10.1007/s10531-013-0607-0

[325] Do TH, Krott M, Juerges N, Bröcher M. Red lists in conservation science-policy interfaces: A case study from Vietnam. Biol Conserv. 2018;226: 101–110. doi: 10.1016/j.biocon.2018.07.016

[326] Do TH, Bröcher M, Krott M. Multiple traps of scientific knowledge transfer: Comparative case studies based on the RIU model from Vietnam, Germany, Indonesia, Japan, and Sweden. Forest Policy Econ. 2020;114: 102134. doi: 10.1016/j.forpol.2020.102134

[327] Funabiki A, Haruyama S, Nguyen VQ, Pham VH, Dinh HT. Holocene delta plain development in the Song Hong (Red River) delta, Vietnam. J. Asian Earth Sci. 2007;30: 518–529. doi: 10.1016/j.jseaes.2006.11.013

[328] Campbell JFE, Fletcher WJ, Hughes PD, Shuttleworth EL. A comparison of pollen extraction methods confirms dense-media separation as a reliable method of pollen preparation. J Quaternary Sci. 2016;31(6): 631–40. doi: 10.1002/jqs.2886

[329] Tobler MW, Carrillo-Percastegui SE, Leite Pitman R, Mares R, Powell G. An evaluation of camera traps for inventorying large- and medium-sized terrestrial rainforest mammals. Anim Conserv. 2008;11: 169–178. doi: 10.1111/j.1469-1795.2008.00169.x

